# Phosphoserine aminotransferase 1 promotes serine synthesis pathway and cardiac repair after myocardial infarction

**DOI:** 10.7150/thno.112077

**Published:** 2025-06-18

**Authors:** Ajit Magadum, Vandana Mallaredy, Rajika Roy, Darukeshwara Joladarashi, Charan Thej, Zhongjian Cheng, Maria Cimini, May Truongcao, Adam Chatoff, Claudia V Crispim, Vagner O C Rigaud, Carolina Gonzalez, Cindy Benedict, Celio X.C. Santos, Nathaniel W. Snyder, Mohsin Khan, Ajay M. Shah, Walter J. Koch, Raj Kishore

**Affiliations:** 1 Aging+Cardiovascular Discovery Center, Lewis Katz School of Medicine, Temple University, Philadelphia, PA 19140, USA.; 2 Department of Cardiovascular Sciences, Lewis Katz School of Medicine, Temple University, Philadelphia, PA 19140, USA.; 3 Department of Surgery, Division of Cardiovascular and Thoracic Surgery, Duke University School of Medicine, Durham, NC, USA.; 4 Center for Metabolic Disease Research, Lewis Katz School of Medicine, Temple University, Philadelphia, PA 19140, USA.; 5 King's College London British Heart Foundation Centre, School of Cardiovascular Medicine & Sciences, United Kingdom.

**Keywords:** cardiac repair, mRNA therapeutics, cardiomyocyte proliferation, apoptosis, oxidative stress, serine synthesis pathway

## Abstract

**Background and Purpose:** The permanent loss of cardiomyocytes (CMs) following myocardial infarction (MI), coupled with the heart's limited regenerative capacity, often leads to heart failure. Phosphoserine aminotransferase 1 (PSAT1) is a protein highly expressed in the embryonic mouse heart but markedly downregulated after birth. Despite its presence in early cardiac development, PSAT1's role in CM proliferation, cardiac physiology, and repair remains unexplored. This study investigates the therapeutic potential of PSAT1-modified mRNA (modRNA) for promoting cardiac repair and improving outcomes post-MI.

**Methods:** Synthetic PSAT1-modRNA was delivered to the hearts of mice post-MI. The study evaluated its effects on CM proliferation and death, scar formation, angiogenesis, and cardiac function. Molecular mechanisms underlying PSAT1's actions were explored, including its regulation of the serine synthesis pathway (SSP), oxidative stress, nucleotide synthesis, and interactions with the YAP1-β-catenin molecular axis. Additionally, SSP inhibition studies were conducted to determine its contribution to CM cell cycle activity and apoptosis.

**Results:** PSAT1 is downregulated during mouse heart development. Cardiac delivery of PSAT1-modRNA induced significant CM proliferation, reduced scar size, and enhanced angiogenesis. Functional analyses revealed improved cardiac performance and survival in PSAT1 injected mice post-MI. Mechanistically, PSAT1 induces the serine synthesis pathway (SSP) in CMs, resulting in increased nucleotide synthesis and reduced oxidative stress, thereby supporting CM proliferation and survival**.** Conversely, SSP inhibition suppressed CM cell cycle activity and triggered apoptosis post-MI. Furthermore, PSAT1 modRNA inhibited CM apoptosis by reducing oxidative stress and DNA damage. At the molecular level, YAP1 transactivated PSAT1, and PSAT1 induced β-catenin nuclear translocation, and is indispensable for YAP1-induced CM proliferation.

**Conclusions:** PSAT1 emerges as a pleiotropic gene critical for favorable cardiac remodeling post-MI through multiple mechanisms, including CM proliferation, SSP activation, inhibition of oxidative stress and cell death, and YAP1-β-catenin pathway modulation. These findings highlight PSAT1's potential as a novel therapeutic target for mRNA-based treatments in ischemic heart diseases, offering a promising avenue for clinical application in cardiac repair.

## Introduction

In 2020, over 19 million global deaths were attributed to CVD, an increase of 18.71% from 2010. The prevalence of CVD increased with age in both males and females 1. Despite the improvement shown in the prognosis of patients with acute MI with the use of available therapies including thrombolysis and urgent coronary revascularizations, a considerable proportion of MI survivors are at risk for developing heart failure. Heart failure remains major health concern in developed world, with hospital admission rates remaining stable over the past decade. Surgical approaches like cardiac transplants and artificial implants aside, current interventional strategies are geared towards providing symptomatic relief. Since the major cause of developing heart failure is the massive loss of cardiomyocytes after MI and since adult cardiomyocytes are refractive to proliferation and renewal, identifying pathways to stimulate endogenous CM regeneration holds substantial clinical promise. Mammalian cardiomyocyte (CM) proliferation rapidly ceases after birth, and the heart further grows by an increase in CM size rather than number 2, 3. Adult zebrafish, newt, and even 1-day-old neonatal mouse and pig can regenerate the heart by inducing CM proliferation post-injury 4-8. Previously, attempts of limited success have been made to transiently reconstitute embryonic signaling in adult hearts, including overexpression of cell cycle-activating genes or induction of novel genes or molecular pathways or cardiac microenvironment 9-26. Recent studies suggest that metabolic pathways play a crucial role in cardiac repair and regeneration 9, 10, 27. Serine synthesis pathway (SSP), which generates serine either through cellular uptake or de novo biosynthesis. Serine serves as a precursor for nucleotides, proteins, and lipids, supports antioxidant defense through glutathione and NADPH production, and provides one-carbon units for methylation reactions. While SSP has been implicated in tissue repair in the liver, skin, and nervous system, its role in cardiac repair and CM proliferation remains largely unexplored. Emerging evidence, however, points to its therapeutic relevance. For example, activation of serine one-carbon metabolism by calcineurin Aβ1 reduces cardiac hypertrophy and improves function 28, and SSP activation has been shown to rescue contractile defects in iPSC-derived cardiomyocytes from dilated cardiomyopathy patients 29. The inhibition of the hippo pathway or YAP1 activation plays a critical role in CM proliferation and cardiac regeneration 30, 31. While Hippo pathway activation known to induce dilated cardiomyopathy 32, 33. YAP1 is regulated at multiple levels in the heart, including transcriptional, post-translational, and epigenetic mechanisms 34-41. However, little is known about the role of YAP1 in metabolism-mediated cardiac regeneration.

Significant challenges exist with current gene delivery methods, including short half-life modes of delivery, and uncontrolled expression that may potentially lead to cardiac hypertrophy and arrhythmia 42, 43. The modified RNA (modRNA) based gene therapy approach is attractive because of modRNAs ability for prompt translation to protein, dose controlled robust yet transient expression pattern, higher RNase resistance ability and no risk of genomic integration or activation of innate immunity 10, 44-49. ModRNA is synthesized with 100% replacement of Uridine by N1-Methylpseudouridine-5'-Triphosphate (1-mψU) 44, 45. As was recently published, modRNA transfection induces transient, pulse-like cognate protein expression *in vitro* (rat neonatal CMs) as well as *in vivo* (mouse adult heart, 7-12 days) and improves cardiac function and repair post-MI 10, 44, 47, 48, 50-53. In this study, we aimed to investigate the role of the novel gene PSAT1 in promoting cardiac repair post-heart injury, which has not been previously explored in heart biology and repair. PSAT1 is highly expressed in the embryonic mouse heart but markedly downregulated after birth. Here, we show that cardiac delivery of PSAT1 modRNA promotes CM proliferation, enhances angiogenesis, reduces fibrosis, and improves cardiac function and survival post-MI. PSAT1 activates the SSP, increasing nucleotide production and reducing oxidative stress. Inhibition of SSP impairs CM proliferation and induces apoptosis. Mechanistically, YAP1 transactivates PSAT1, which stabilizes β-catenin and drives CM cell cycle entry. Our findings identify PSAT1 as a pleiotropic regulator of cardiac repair and a promising candidate for mRNA-based therapies in ischemic heart disease.

## Methods

All data presented in this study are freely available from the corresponding author upon any reasonable request.

### Mice

All animal procedures were performed under protocols approved by the Temple University Animal Care and Use Committee and the NIH Guide for the Care and Use of Laboratory Animals. C57BL6J mice strains, male and female, were used. Different modRNAs (100 μg/heart) were injected directly into the myocardium in open-chest surgery. Three to ten animals were used for each experiment. For mice survival, C57BL6J mice (10-12 weeks old) were treated with Luc or PSAT1 modRNAs (n = 10) post-induction of MI and were followed for the indicated time. Deaths were monitored and documented over time. Tamoxifen-inducible CM-restricted MADM mice (α-MHC-MADM mice) were generated by crossing MADM mice (Jackson laboratory) to B6.FVB (129)-A1*cf.*Tg(Myh6-cre/Esr1∗)1Jmk/J (Cat no. 005657) mice to get α-MHC-MADM mice (These mice were a generous gift from Dr. Khan's laboratory, co-author in the manuscript) 54. Tamoxifen (Sigma-Aldrich) was dissolved in sesame oil at 10 mg/mL as stock solution and mixed well. To induce Cre activation, tamoxifen was administered to mice by intraperitoneal (IP) injection for 5 times every 48 h (48h interval between administrations) with a 40 mg/kg body weight for adult mice 54. The tissues were harvested at the indicated time for study.

### Synthesis of modRNA

ModRNAs were transcribed *in vitro* from plasmid templates (ordered from GeneScript). See complete list of modRNA sequence or open reading frame sequences used to make the modRNA for this study (Table 1). using a customized ribonucleotide blend of cap analog, CleanCap (Cap1) (6 mM, TriLink Biotechnologies), guanosine triphosphate (1.5 mM, Life Technology), adenosine triphosphate (7.5 mM, Life Technology), cytidine triphosphate (7.5 mM, Life Technology) and N1-Methylpseudouridine-5'-Triphosphate (7.5 mM, TriLink Biotechnologies) as described previously 10, 44. The mRNA was purified using RNA isolation kit (Qiagen) and quantitated by Nanodrop (Thermo Scientific), precipitated using ethanol and ammonium acetate, and resuspended in water or 10 mM TrisHCl, 1 mM EDTA.

### ModRNA transfection

We previously standardized the concentration and delivery methods for modRNA transfection in both in vitro and in vivo cardiac models 10, 44, 46, 49, 55. For in vivo applications, transfection was performed as described in prior studies using a sucrose-citrate buffer. This buffer consisted of 10 μL of sucrose (0.3 g/mL in nuclease-free water), 10 μL of citrate (0.1 M, pH 7; Sigma), and 10 μL of modRNA (varied concentrations) diluted in saline to a total volume of 30 μL. The transfection mixture can be stored at -80 °C. Immediately following LAD ligation, mice underwent open-chest surgery to receive intra-myocardial injections of modRNA (100 μg in 30 μL) at three peri-infarct sites: two mid-anterior and one apical-anterior. ModRNA expression in heart tissue was analyzed 24 h post-injection.

For *in vitro* transfection, RNAiMAX transfection reagent (Life Technologies) was used following the manufacturer's protocol. A mixture of 2.5 μg of modRNA (PSAT1 or Luc) and RNAiMAX reagent was prepared and applied to cultured cells in an each well of 24-well plate 20 min later. After transfection, cells were fixed or harvested for downstream analyses.

### Neonatal rat cardiomyocytes or fibroblast isolation

CMs from or 2-3-day-old neonatal rat's (Sprague Dawley rats) heart were isolated after decapitation of pups as previously described (Jackson) 10. We used multiple rounds of digestion with 0.14-mg/mL collagenase II (Invitrogen). After each digestion, the supernatant was collected in Horse serum (Invitrogen). Total cell suspension was centrifuged at 300 g for 5min. Supernatants were discarded, and cells were resuspended in DMEM (GIBCO) medium with 0.1 mM ascorbic acid (Sigma), 0.5% Insulin-Transferrin-Selenium (100×), penicillin (100 U/mL) and streptomycin (100 μg/mL). Cells were plated in plastic culture dishes for 90 min until most of the non-myocytes attached to the dish (and mostly contains fibroblasts) and myocytes remained in suspension. Myocytes were then seeded at 1 × 10^5^ cells/well in a 24 well plate. Isolated CMs were incubated for 48 h in DMEM medium containing 5% horse serum plus Ara c. Fibroblasts were seeded at 1 × 10^5^ cells/well in a 24 well plate in DMEM (GIBCO) medium and penicillin (100 U/mL) and streptomycin (100 μg/mL) for 48 h. After incubation, NRVMs or fibroblasts were transfected with different doses of different modRNAs, or siRNA as described in the text. PH755 a small molecule inhibitor of phosphoglycerate dehydrogenase (PHGDH) at 10µM was used to inhibit SSP. To study CM apoptosis or analyze TUNEL^+^ CMs, NRVMs were treated with or without PH755 in presence or absence of PSAT1 modRNA. Cells were kept in hypoxia chamber for 24 h.

### Mouse MI model and histology

All experimental and surgical procedures with mice were performed in accordance with protocols approved by Institutional Animal Care and Use Committees (IACUC) at Temple University and the NIH Guide for the Care and Use of Laboratory Animals. C57BL6J or CM-specific MADM mice (8-12 weeks- old) were anesthetized with 2% isoflurane (inhalation) throughout the surgical procedure. MI was induced in mice by permanent ligation of the LAD, as previously described 56. Briefly, the left thoracic region was shaved and sterilized. After intubation, the heart was exposed through a left thoracotomy. A suture was placed to ligate the LAD. The thoracotomy and skin were sutured closed in layers. Excess air was removed from the thoracic cavity, and the mouse was removed from ventilation when normal breathing was established. Postoperative analgesia buprenorphine (0.06 mg/kg) was given once subcutaneously. To study the effect of modRNA on cardiovascular outputs after MI, modRNA (100 μg/heart) was injected into the infarct zone immediately after LAD ligation 10. In all experiments, the surgeon and echo analysis person were blinded to the treatment group. For examination of heart histology, hearts were collected at the end of each study by cervical dislocation and pneumothorax based on methods consistent with the recommendation of the American Veterinary Medical association (AVMA) Guidelines for the Euthanasia of Animals. Heart shortly washed in PBS, weighed, and fixed in 4% PFA at 4 °C overnight. The heart sections were processed for histological evaluation employing immunostaining (see below) or histological scar staining using Masson's trichrome staining kit (Sigma), according to standard procedures and scar size was measured as showed before. Measuring the ratio of heart weight to body weight was done using a scale. This ratio was calculated as the heart tissue weight relative to the total mouse body weight in grams (g).

### Cardiomyocyte specific mosaic analysis with double markers (α-MHC-MADM mice)

To analyze the CM division *in vivo* post-MI, we used CM-specific mosaic analysis with double markers (MADM) mice generated as described previously 54, 57. The CM-specific transgenic mice were generated by crossing MADM mice (Jackson laboratory) to B6.FVB (129)-A1*cf.*^Tg(Myh6-cre/Esr1∗)^1Jmk/J (Cat no. 005657) mice to get α-MHC-MADM mice (These mice were a generous gift from Dr. Mohsin Khan Laboratory, co-author in the manuscript). In the MADM system, because of intrachromosomal Cre-loxP recombination after S phase of the cell cycle, the daughter cells will get either labeled (single-colored) with GFP (Green) or RFP (Red). The MADM single labeling can only be attained by the end of the cell cycle through cytokinesis. The inducible MADM system warrants temporal control of recombination and offers direct evidence for cell division. The α-MHC driven MADM mice will allow us to analyze the CM division carefully without the interference of non-CM proliferation and non-CM-CM cell fusion. In acute MI setting, 8-12-week-old α-MHC-MADM mice were injected immediately after MI with Luc or PSAT1 modRNA. To evaluate the % single color of total labeled (Cre activated cells) CMs, mice were harvested after 4 weeks post-MI and immunostained.

### Immunostaining of heart sections following modRNA treatment

Mice hearts were harvested and weighted. Next, hearts were fixed in 4% PFA/PBS overnight on a shaker and then washed with PBS for 1 h and incubated in 30% sucrose at 4 °C for 12-18 h. Finally, hearts were fixed in OCT and kept at -80 °C 10. Transverse heart sections (6-8 μM) were done using a cryostat machine. Frozen heart sections were rehydrated in PBS for 5-10 min, pursued by permeabilization with PBS with 0.1% triton X100 (PBST) for 7-8 min. Heart sections were then treated with 3% H2O2 for 5 min and 3 times washed with PBST for 5 min each. Then samples were blocked with PBS + 10% Donkey normal serum + 0.1% Triton X100 (PBSST) for 1 h. Next, the primary antibodies (see a complete list of primary antibodies used for this study in Table 2) diluted in PBSST were added and incubated overnight at 4 °C. Slides were further washed with PBST (5 min each for 3 times) followed by incubation with a secondary antibody (Invitrogen, 1:200) diluted in PBST for 90 to 120 min at room temperature. Next, slides were washed with PBST (3 times for 5 min each) and stained with DAPI or Hoechst 33342 (1 μg/mL) diluted in PBST for 7 min. After 3 washes with PBST for 7 min each and one time with tap water for 5 min, slides were mounted with mounting medium (VECTASHIELD) for imaging 10. Stained slides were stored at 4 °C. All staining was performed on 4-8 hearts/group, with 2-3 sections/heart. For immunostaining with wheat germ agglutinin (WGA) for CM size quantification, images at 40X magnification were captured, and ImageJ was used to determine CM surface area. Quantitative analyses involved counting of multiple fields from 5-8 mice hearts per group and 3-4 sections/heart (~50 cells per microscope field assessed, to a total ~250 cells per sample). For BrdU immunostaining, BrdU (1mg/mL, Sigma) was added to the drinking water of adult mice (2-3-month-old) for 7 days before collecting the hearts. After heart section permeabilization, DNA denaturation was obtained by incubating 10 min in 1 M HCl on ice and 20 min in 2 M HCl at 37 °C. Sections were further incubated with 0.1 M sodium-borate buffer pH 8.4 for 12 min at room temperature, washed three times with PBS and then blocked for 1 h in 10% donkey normal serum. BrdU positive CMs were counted in multiple fields from three independent samples per group and 3 sections/heart. The total number of CMs counted was ~1-1.5×10^3^ CMs per heart section. TUNEL immunostaining of heart sections was performed according to the manufacturer's recommendations (*In-Situ* Cell Death Detection Kit, Fluorescein, Cat# 11684795910, Roche) 10. For immunostaining of NRVMs were fixed on coverslips with 3.7% PFA for 15 min at room temperature. 0.5% Triton X in PBS for 10 min at room temperature were used cell permeabilization. Next cells were blocked with 5% normal donkey serum + 0.5% Tween 20 for 30 min. Cells were incubated with primary antibodies in a humid chamber for 1 h at room temperature. Then coverslip is incubated with corresponding secondary antibodies conjugated to Alexa Fluor 488 and Alexa Fluor 555, and nuclei visualization by Hoechst 33342 (all from Invitrogen) 10. The fluorescent images were taken on fluorescent microscopy at 10X, 20X, and 40X magnification.

### Cardiac YAP1 knockdown

Mice were injected with AAV9-GFP or AAV9-YAP1-shRNA (ordered from GeneCopoeia) through retro-orbital injection 7 days before LAD. We injected total of 5 x 10^11^ viral genomes (50 μl total volume delivered) per mice. The level of YAP1 inhibition was determined at mRNA level by qPCR 14 days post-injection.

### Western blot analysis

We isolated total protein from the respective cells or tissues at a given time point. In brief, equal amounts of protein were resolved by SDS-PAG Electrophoresis system in 4%-15% Mini-PROTEAN TGX stain-free gels (Bio-Rad) and blotted onto Nitrocellulose Membranes (Bio-Rad). The membranes were blocked in Intercept blocking buffer (LI-COR) in 1:1 dilution with PBS for 1 h at room temperature and then washed with PBST (1% Tween 20) (PH 7.4) for 10 min. Primary antibodies diluted in the above blocking buffer were used overnight at 4^0^C. We used anti-pSer9GSK3 β (1:1,000, Cell Signaling, #9336), anti-GSK3 β (1:1,000, Cell Signaling, #9315), anti-PSAT1 (1:1,000, Invitrogen, PA5-22124) and anti-β-actin (1:1000, Santa Cruz, #sc-47778) antibodies. The next day membranes were washed three times with PBST 10 mins (3 times). Anti-rabbit and anti-mouse IRDye 800CW secondary antibodies were purchased from LI-COR and were applied for 1 hr at room temperature. The membranes were washed with PBST 10 min three times, and Antigen or antibody complexes were visualized and quantified using Odyssey Fc Imaging System (LI-COR Biosciences, model number 2800).

### RNA isolation and gene expression profiling using Real-Time PCR

Total RNA was isolated using Trizol RNA isolation and RNAeasy (Qiagen) then reverse transcribed with High-Capacity cDNA Reverse Transcription Kit (Applied biosystems), according to the manufacturer's instructions. Real-time qPCR analyses of samples were performed on a StepOnePlus™ Real-Time PCR System using an SYBR Green (Applied Biosystems). The primer sequences were synthesized by IDT (Table 3). Data were normalized to 18s expression, where appropriate (endogenous controls). Fold changes in gene expression were determined by the ∂∂CT method and were presented relative to internal control.

### Chromatin immunoprecipitation

Neonatal rat CMs were transfected with Luc or YAP1 modRNA and cells were fixed after 24 h with 1% formaldehyde for 15 min and quenched with 0.125 M glycine, and sent to Active Motif Services (Carlsbad, CA) to be processed for ChIP-qPCR. In brief, chromatin was isolated by the addition of lysis buffer, followed by disruption with a Dounce homogenizer. Lysates were sonicated and the DNA sheared to an average length of 300-500 bp. Genomic DNA (Input) was prepared by treating aliquots of chromatin with RNase, proteinase K and heat for de-crosslinking, followed by SPRI bead clean up (Beckman Coulter). Pellets were resuspended and the resulting DNA was quantified on a ClarioStar spectrophotometer (BMG Labtech). Extrapolation to the original chromatin volume allowed quantitation of the total chromatin yield.

An aliquot of chromatin (30 ug) was precleared with protein A agarose beads (Invitrogen). Genomic DNA regions of interest were isolated using 5 ug of antibody against YAP1 (US Biological, catalog number Y1200-01D). Complexes were washed, eluted from the beads with SDS buffer, and subjected to RNase and proteinase K treatment. Crosslinks were reversed by incubation overnight at 65 ℃, and ChIP DNA was purified by phenol-chloroform extraction and ethanol precipitation.

Quantitative PCR (qPCR) reactions were conducted in triplicate on specific genomic regions using SYBR Green Supermix (Bio-Rad, Cat # 170-8882) on a CFX Connect™ Real Time PCR system. The resulting signals were normalized for primer efficiency by conducting QPCR for each primer pair using Input DNA (Primer sequence is in Table 4).

### RNA interference

For siRNA knockdown, CMs were transfected 48 to 72 h after seeding by lipofectamine RNAiMAX kit (Invitrogen) with validated siRNAs or All Stars Negative Control siRNA (Qiagen) (100 nM) and washed after 5 h. Gene expression inhibition was verified by antibody staining after 72 h. siRNA sequences: β-catenin-5'-UAGUCGUGGGAUCGCACCCTG-3', PSAT1-5'-AUGUCCAUGACGUAGAUGC-3'.

### ^13^C_6_ isotopic tracing and liquid chromatography high resolution mass spectrometry

250,000 NRVMs (P2-P3) were cultured in a DMEM medium and transfected with Luc or PSAT1 modRNA. After 12 h, the medium was replaced with DMEM containing ^13^C_6_ Glucose. The samples were collected at 10 min, 8 h, and 24 h by washing the cells or culture plates twice with ice-cold 0.9% NaCl solution, followed by the addition of 1 mL of -80 °C 80% methanol in water to extract metabolites. Metabolites were then extracted by scraping the cell culture plates, transfer to 1.5 mL Eppendorf tubes, vortex mixing for 1 min, chilling to -80 for 2 h, centrifugation of the insoluble debris for 10 min at 18,000 x g at 4 °C and then evaporating the clarified supernatant to dryness Samples were resuspended in 50 μL of 95:5 water: methanol. Liquid chromatography- high resolution mass spectrometry analyses were performed on 2 uL injections of samples onto a Q Exactive Orbitrap mass spectrometer operating in polarity switching mode (Thermo Scientific) coupled to a Vanquish UPLC system (Thermo Scientific). A Sequant ZIC-HILIC column (2.1 mm i.d. × 150 mm, Merck) was used for the separation of metabolites. The flow rate was 150 μL/min. Buffers consisted of 100% acetonitrile for A and 0.1% NH4OH/20 mM CH3COONH4 in water for B. Gradient ran from 85 to 30% A in 20 min, followed by a wash with 30% A and re-equilibration at 85% A. Metabolites were identified based on exact mass within 5 ppm and standard retention times. Relative metabolite quantitation was performed based on the peak area for each metabolite. Analysts were blinded to sample identity during extraction and data analysis.

### Serine synthesis pathway inhibition by NCT-503

NCT-503 (obtained from Sigma) was formulated in a vehicle consisting of 5% ethanol, 35% PEG 300 (Sigma), and 60% of an aqueous 30% hydroxypropyl-β-cyclodextrin (Sigma) solution. The prepared vehicle or NCT-503 (at a dosage of 30 mg/kg for each mouse) was administered intraperitoneally once daily, initiated one day prior to MI and continued throughout the experiment. The dosage was adjusted based on the weight of the mouse, ensuring that the volume of injection did not exceed 150 µL.

### Cardiomyocyte number analysis

In the acute MI models, mice aged 8-12 weeks received immediate injections of either Luc or PSAT1 modRNA following MI. After a 30-day interval, adult cardiomyocytes were isolated from these mice using Langendorff method, and the number of cardiomyocytes per heart was calculated. The isolated cardiomyocytes were subsequently fixed and immunostained with the cardiomyocyte marker α-sarcomeric actinin and DAPI to assess nucleation.

### HPLC measurements of ROS

ROS production in heart tissue was measured using HPLC for dihydroethidium (DHE) oxidation products 10. Immediately after harvesting hearts, a 20 mg segment was cut into small pieces and incubated with DHE (100 μM) in PBS containing DTPA (100 μM) at 37 °C for 30 min. The sample was washed with PBS/DTPA, extracted with acetonitrile (500 μl) and briefly sonicated (3x30 sec, 8W). After spinning (13,000Xg, 10 min at 4 °C), the supernatant was collected and dried under vacuum, and pellets stored at -20 °C in the dark until analysis. Samples were resuspended in 120 μl PBS-DTPA and injected into the HPLC system. The superoxide-specific DHE oxidation product, 2-hydroxyethidine (EOH), was quantified by comparison of the peak signal between samples and standard solutions under identical chromatographic conditions and expressed as EOH/mg tissue.

### HPLC measurements of glutathione

The level of glutathione (GSH) in heart tissue was measured by an HPLC method with electrochemical detection, as described previously 10. Heart samples were homogenized in a 100 mM acetate buffer (pH 5.4, containing 10 µM DTPA). A small aliquot was used to quantify total protein. The remaining sample was deproteinized with 10% TCA, centrifuged at 10,000 g for 5 min at 4^0^C, the supernatant filtered (0.45 µm) and stored at -80^0^C. Samples were applied to a C18 column (Phenomenex C18, 3 μm, 150 × 4.6 mm) at 0.5 mL.min^-1^ using isocratic mobile phase solution (25 mM NaH_2_PO_4_, 1 mM 1-octane sulfonic acid, 6% acetonitrile, pH 2.6, with phosphoric acid). GSH levels were quantified by comparison to standards subjected to the same HPLC conditions. Glutathione disulfide (GSSG) levels were indirectly determined by its enzymatic reduction with glutathione reductase (0.6 U/mL plus 0.2 mg/mL NADPH) for 30 min. The reaction was stopped by adding 5% TCA on ice. The GSH levels were normalized by the amount of protein. GSSG quantification was achieved by the difference between total reduced GSSG to total GSH in each sample.

### Serine level analysis

The plasma sample (~100µl) was centrifuged at 10,000 g for 5 min at 4 °C, and the supernatant was carefully transferred to a new microfuge tube. For the heart tissue, we quickly homogenized 10 mg of tissue on ice using 100 µL of ice-cold Serine Assay Buffer. The homogenate was then centrifuged at 15,000 g for 10 min at 4 °C, and the resulting supernatant was transferred to a fresh microfuge tube. Subsequent steps followed the manufacturer's instructions, and the fluorescence of all samples was measured at an excitation wavelength of 535 nm and an emission wavelength of 587 nm in endpoint mode.

### Statistical analysis

Statistical significance was determined by Unpaired two-tailed t-test, One-way ANOVA, Bonferroni post hoc test or Log-rank (Mantel- Cox) test for survival curves as detailed in respective figure legends. A pValue < 0.05 was considered statistically significant for all analyses. All graphs represent average values, and values were reported as mean ± standard error of the mean. Unpaired two-tailed t-test was used for comparing two independent groups, assuming a normal distribution of data. It was applied where appropriate for continuous variables. One-way ANOVA was used to compare means across three or more groups, followed by the Bonferroni post hoc test to adjust for multiple comparisons. This approach helped identify specific group differences while controlling for type I errors. This test was used to compare survival curves and assess differences in survival rates between groups. For quantification of parameters such as the number of luminal structures (CD31), WGA, 8-OHG, CD45, vimentin, β-catenin, TUNEL-positive nuclei, nucleation, MADM (lineage tracing), BrdU, or pH3-positive cardiomyocytes, results were obtained from at least 3-5 heart sections per heart and a sample size of 3-9 mice giving plenty N number to apply statistical methods. The additional information on number and age of mice analyzed is specified in the respective figure legends. This ensures robust and representative sampling across biological replicates.

## Results

### PSAT1 expression is downregulated in postnatal mouse hearts

**Figure 1:**
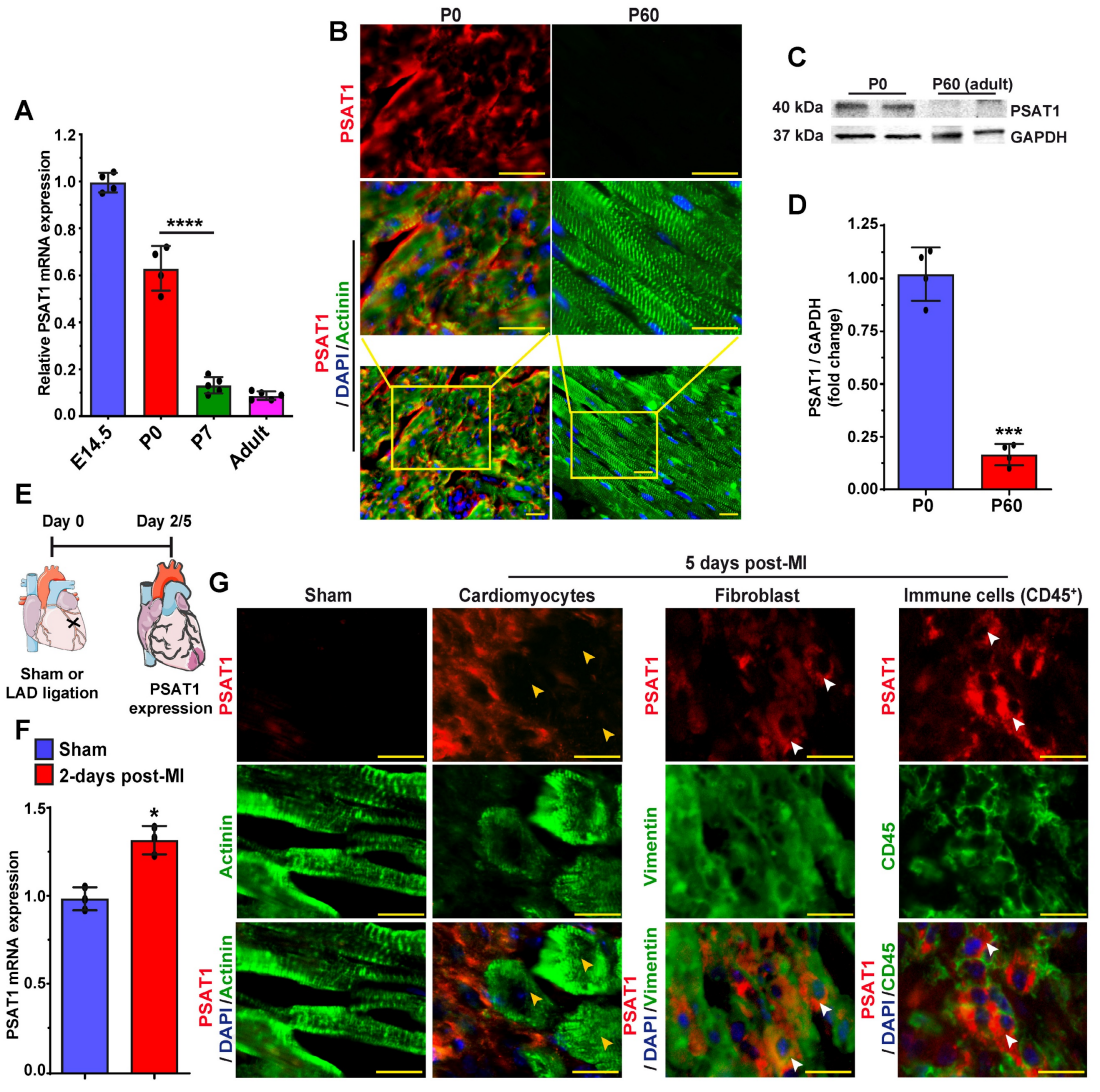
**PSAT1 expression is downregulated during mouse heart development**. A. QPCR analysis of PSAT1 mRNA expression during mouse heart development (E14.5 and Po, n = 4; P7 and Adult, n = 5), showing PSAT1 expression downregulate during mouse heart development. **B.** Representative images of PSAT1 expression (Red), CM-specific marker α-sarcomeric actinin^+^ (Green) and DAPI (blue) in myocardium during mouse heart development (n = 5). **C.** Western-blot analysis of PSAT1-expression Day 0 mice and 60-day old mice heart. **D.** Quantitative analysis of differential protein expression in C (n = 2). **E.** Experimental scheme for PSAT1 expression in myocardium 2 or 5-days post-MI. **F.** QPCR analysis of PSAT1 mRNA expression 2-days post-MI (n = 3). **G.** Representative images of PSAT1 expression (Red), CM-specific marker α-sarcomeric actinin^+^ (Green) or fibroblast -specific marker vimentin^+^ (Green) or endothelial cell-specific marker CD31^+^ (Green), and DAPI (blue) showing PSAT1 expression is induced mostly in non-cardiomyocytes, but not in cardiomyocytes in myocardium 5-days post-MI (n = 5). Unpaired two-tailed t-test for A, D and F. ***, P < 0.001, *, P < 0.05. Scale bar = 20 μm (B and G).

PSAT1 is an enzyme involved in the serine synthesis pathway, where it converts 3-phosphohydroxy pyruvate in the presence of glutamate to 3-phosphoserine and α--ketoglutarate. PSAT1 is predominantly expressed in brain and liver 58. The role and function of PSAT1 in CMs, heart biology, diseases, and repair have never been studied before. Therefore, first, we analyzed the developmental expression pattern of PSAT1 in mouse embryonic, neonatal, and adult hearts. PSAT1 mRNA was abundant during embryonic development; however, its expression was significantly downregulated by postnatal day 7 with minimal expression in adult heart (Figure 1A). Similarly, PSAT1 protein was mainly localized in cardiomyocyte cytoplasm during embryonic development and was at undetectable levels in adult mice (Figure 1B). Western blot analysis also showed significant decrease in PSAT1 expression in adult mouse heart (Figure 1C-D). While cardiac expression of PSAT1 was moderately increased post-MI 59, but it was mostly detected in non-CMs like immune cells (CD45^+^ cells) and fibroblasts, but not in cardiomyocytes (Figure 1E-G).

### PSAT1 modRNA induces cardiomyocyte proliferation post-MI

**Figure 2:**
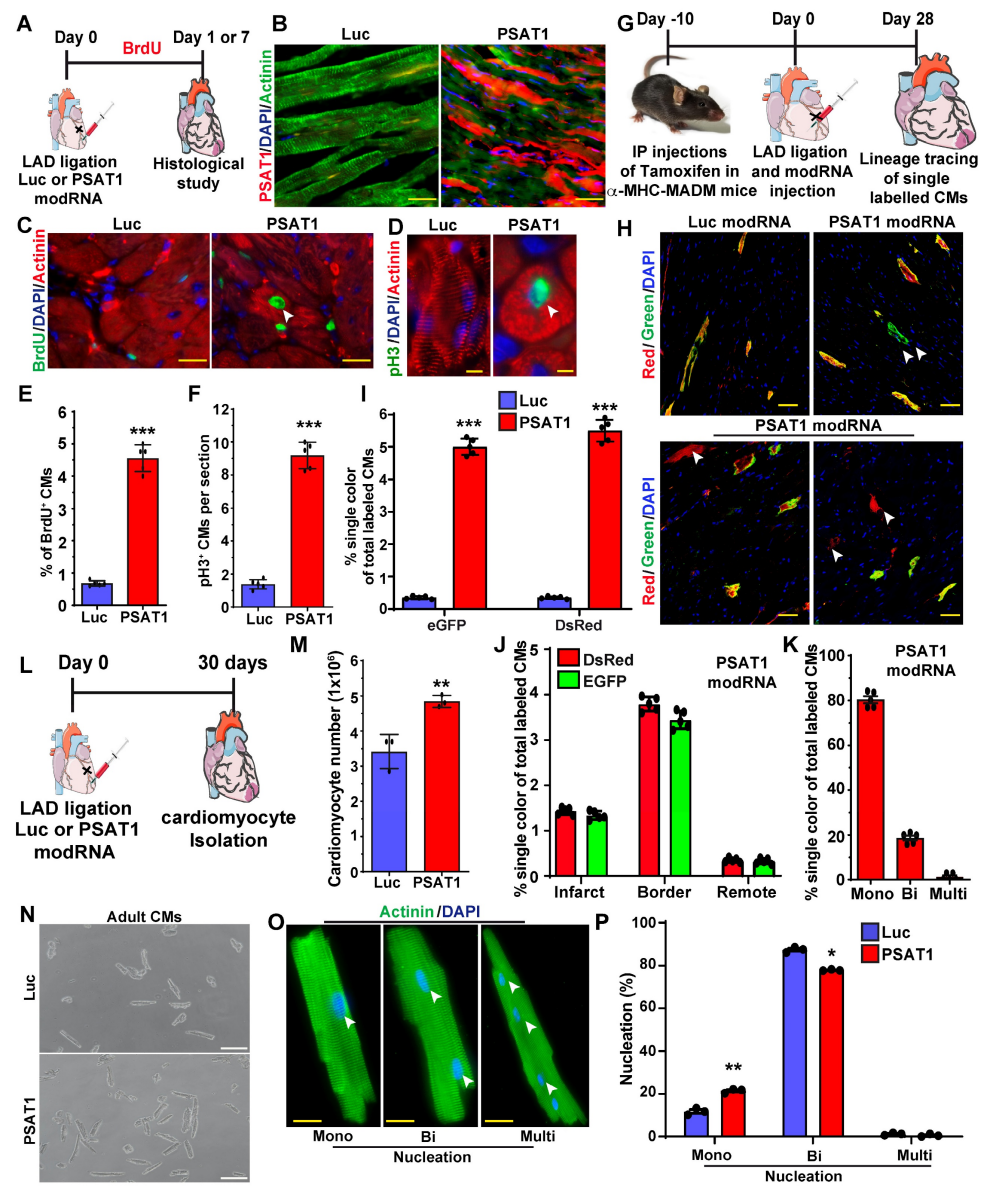
PSAT1 modRNA induces CM proliferation post-MI. A. Experimental scheme for PSAT1 modRNA expression in myocardium post-MI and its effect on CMs cell cycle marker expression after Luc or PSAT1 delivery to the heart. **B.** Representative images of PSAT1 modRNA expression (Red), (CM specific marker α-sarcomeric actinin in Green) 24 h. post-MI after Luc or PSAT1 modRNA injection. **C-D.** Representative images of BrdU^+^ (Green) (C) or pH3^+^ (Green) (D), CM-specific marker α-sarcomeric actinin^+^ (Red) and DAPI (blue) post-MI. **E-F.** Quantitative analysis of BrdU^+^ or pH3^+^ CMs seven days post-MI and Luc or PSAT1 modRNA treatment (n = 5), showing induced cardiomyocyte cell cycle activity. **G**. Experimental timeline to trace proliferating CMs using α-MHC-MADM mice **H.** Representative images of single-color-(Green (eGFP^+^/DsRed^-^), Red (eGFP^-^/DsRed^+^)) or double- color- (Yellow (eGFP^+^/DsRed^+^) labeled CMs 28 days post MI and injection of Luc or PSAT1 modRNA in a α-MHC-MADM mice. **I.** Quantification of single-color CMs (Red or Green) amongst total labeled CMs 28 days post MI (n = 5) suggesting induced CM cell division after PSAT1 modRNA delivery compared to Luc. **J.** Distribution of single-color CMs in the heart 28 days post MI (n = 5, infarct, border, or remote area). **K.** Quantification of CM nucleation, positive for single color (n = 5, mono, bi or multi) 28 days post MI. **L.** Experimental scheme for PSAT1 modRNA expression in myocardium 30 days post-MI and its effect on CM numbers and nucleation levels. **M.** Quantitative analysis of CM numbers (n = 3) showing PSAT1 modRNA significantly induced CM number post-MI. **N.** Representative images of CM numbers 30 days post-MI after Luc or PSAT1 modRNA injection. **O.** Representative images of α-sarcomeric actinin (Green) and DAPI (blue) to show nucleation (Mono, bi and multinucleation) in CMs 30 days post-MI after Luc or PSAT1 modRNA injection. **P.** Quantitative analysis of O (CM nucleation) (n = 3). Unpaired two-tailed t-test for E-F, I-K, M and P ***, P < 0.001, **, P < 0.01*, P < 0.05. Scale bar = 50 μm (B and C), 10 μm (D and O), 100 μm (H and N).

To study whether exogenous delivery PSAT1 in neonatal rat ventricular CMs (NRVM) or adult CMs post-MI induces CM cell cycle, we used modRNA delivery approach to globally express the PSAT1 *in vitro* and in the post-MI hearts as described earlier 10, 44, 47, 48, 50-52. We designed and chemically synthesized the PSAT1 modRNA *in vitro*. The PSAT1 modRNA expression in NRVM was achieved in hours and lasted up to several days Figure S1A-B) as also shown before that the transient nature of modRNA expression (10, 44, 45, 48, 49, 53. Next, we determined the effect of PSAT1 modRNA on the CM cell cycle after 3 days and found that PSAT1 modRNA significantly induced CM mitosis (pH3^+^), and increased CM number by 2-fold in 5 days Figure S1C-E). To study whether the PSAT1 modRNA can induce the CM cell cycle in the adult mice after MI, we delivered 100µg of PSAT1 or luciferase (Luc) modRNA into the infarct border zone post-MI and observed robust PSAT1 protein expression within 24 h (Figure 2A-B). The immuno-staining analysis showed PSAT1 modRNA induced multiple markers of CM cell cycle and proliferation, including a significant increase in CMs positive for BrdU, pH3 (mitosis marker), at day 7 post-MI compared to Luc modRNA (Figure 2C-F). We also found that PSAT1 modRNA induced the expression of positive cell cycle markers (CDK1, CDC20, CCND2, CCNB1 and cMYC) while inhibiting the negative regulators of cell cycle markers (p21 and p27) compared to Luc modRNA in NRVMs and post-MI (Figure S1F and Figure S2A-B). Furthermore, we used CM-specific MADM mice (α-MHC-MADM mice; lineage-tracing model based on Cre-recombinase dependent mosaic analysis with double markers (MADM) mice) to analyze whether PSAT1 induce true CM cell division post-MI. Myocardial delivery of PSAT1 modRNA increased the percentage of single colored CMs (green or red positive) compared to the total labeled CMs significantly, mainly in the infarct border zone, 1-month post-MI (Figure 2G-K). We also found that most of the single colored (green or red positive) CMs of total labeled CMs were mononucleated (Figure 2K). These results demonstrate that PSAT1 modRNA delivery to the heart induces CM proliferation post-MI. Moreover, we conducted cardiomyocyte number analysis by isolating cardiomyocytes using the Langendorff method 30 days post-MI and modRNA injections. Our findings reveal that PSAT1 modRNA significantly induces CM number in the heart compared to mice injected with Luc modRNA (Figure 2L-N). Additionally, PSAT1 modRNA leads to an increase in the number of mononucleated CMs and a reduction in binucleated CMs post-MI (Figure 2O-P). Overall, the data strongly suggest that PSAT1 modRNA significantly promotes CM proliferation and increases their number post-MI. Previous studies utilizing lineage-tracing analysis in cardiomyocyte-specific MADM mice demonstrated that newly generated or divided cardiomyocytes successfully integrate into the existing myocardium (57. This integration was evident through connexin 43 staining, which revealed electrochemical coupling between labeled and unlabeled cells, indicating that daughter cells can establish functional connections with mature cardiomyocytes. Furthermore, we investigated the direct effects of PSAT1 overexpression on endothelial cell, fibroblast, and immune cell proliferation. Using our neonatal rat cardiac cell culture platform, we transfected cells with Luc or PSAT1 modRNA Figure S3A). Our results indicate that PSAT1 has no significant effect on pH3^+^ fibroblasts, whereas it significantly induces ki67^+^ endothelial cells (Figure S3B-E). Additionally, we analyzed the number of immune cells (CD45^+^) in the infarct and scar area of mouse hearts 7 days post-MI and modRNA injection. Our analysis revealed a non-significant change in CD45^+^ cell count in hearts treated with PSAT1 modRNA compared to those treated with Luc modRNA (Figure S3F-H).

### Myocardial delivery of PSAT1 modRNA improves cardiac function and mice survival post-MI

**Figure 3:**
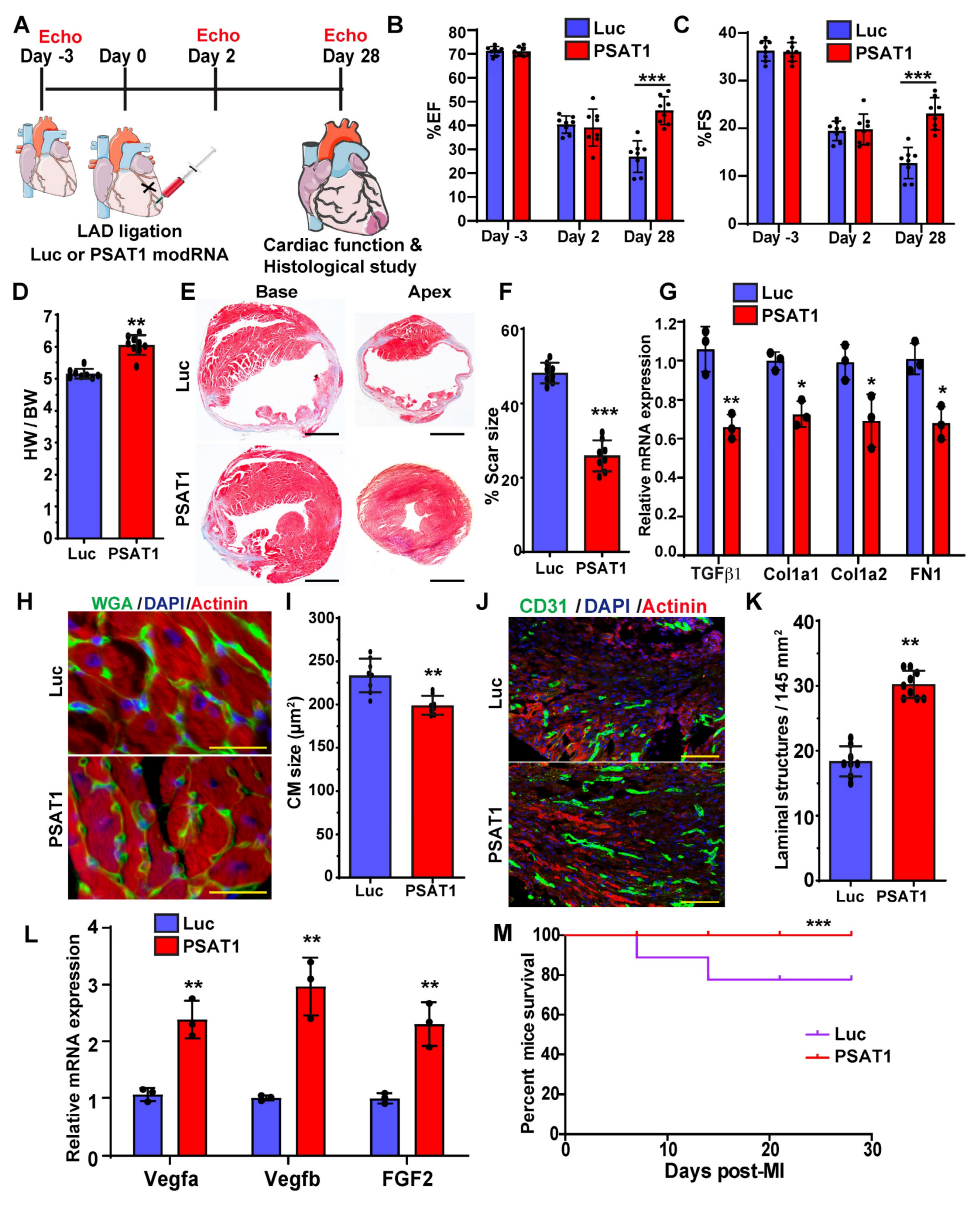
** PSAT1 modRNA delivery improves cardiac function post-MI. A.** Experimental timeline to evaluate cardiac function in MI mouse model 28 days post-MI. **B.** Echo evaluation of percentage of ejection fraction (%EF) (Luc modRNA, n = 8; PSAT1 modRNA, n = 9). **C**. Echo evaluation of percentage fractioning shorting (%FS) post-MI (Luc modRNA, n = 8; PSAT1 modRNA, n = 9). **D.** Heart weight to body weight ratio (Luc modRNA, n = 8; PSAT1 modRNA, n = 9). **E.** Representative Masson trichrome staining to evaluate scar size 28 days post-MI. **F.** Quantification of scar size (Luc modRNA, n = 8; PSAT1 modRNA, n = 9). **G.** QPCR analysis of fibrosis marker (TGFβ1, Cola1, Cola2, and FN1) expression post-MI (n = 3). **H.** Representative images of WGA staining of heart after treatment with Luc or PSAT1 modRNA 28 days post-MI. **I.** CM size quantification by WGA in H (Luc modRNA, n = 8; PSAT1 modRNA, n = 9). **J.** Representative images of CD31^+^ staining of heart sections after Luc or PSAT1 modRNA expression. **K.** Quantification of Capillaries per 145 mm^2^ (Luc modRNA, n = 8; PSAT1 modRNA, n = 9).** L.** QPCR analysis of angiogenesis marker (Vegfa, Vegfb and FGF2) expression post-MI (n = 3). **M**. Long-term survival curve, after MI, for mice injected with Luc or PSAT1 modRNAs, (n = 10). Unpaired two-tailed t-test for (B-C, D, F-G, I, K-L), Montel-Cox log-rank test (M). ****, P < 0.0001, ***, P < 0.001, **, P < 0.01, *, P < 0.05, N.S, Not Significant. Scale bar = 500 μm (E), 100 μm (H and J).

To study the impact of myocardial delivery of PSAT1 modRNA on cardiac remodeling and function post-MI, we analyzed cardiac function and structure 28 days after PSAT1 modRNA delivery (Figure 3A). Echocardiography studies showed that prompt myocardial delivery of PSAT1 modRNA significantly improved the %EF (Ejection Fraction) and %FS (Fractional Shortening) post-MI compared to Luc modRNA (Figure 3B and C). There were also post-MI improvements in LVIDd and LVIDs (Left ventricular internal diameter end-diastole and end-systole) in PSAT1 modRNA-treated mice compared to control. Further, PSAT1 modRNA expression significantly increased LV end-diastolic or systolic posterior wall thickness (Figure S4A-E). The heart weight to body weight (HW/BW) ratio was also significantly increased (Figure 3D). Examination of Masson's trichrome staining of mouse heart demonstrated marked reduction in scar size among mice injected with PSAT1 modRNA, as opposed to those administered Luc modRNA (Figure 3E-F). To delve into the potential impact of PSAT1 on fibrosis, an analysis of fibrosis markers such as TGFβ1, Cola1, Cola2, and FN1 was conducted, revealing a substantial downregulation in all these markers (Figure 3G). Concurrently, visualization of heart sections via wheat germ agglutinin (WGA) staining showcased a significant reduction in cardiomyocyte (CM) size (near infarct-border area) in mice subjected to PSAT1 modRNA injection compared to Luc (Figure 3H-I). This suggests a surge in new muscle formation, not predominantly through pronounced cardiac hypertrophy, but rather due to stimulated CM proliferation. Notably, an enhancement in capillary density post-myocardial infarction (MI) was observed following PSAT1 modRNA injection (Figure 3J-K). Further exploration into this phenomenon uncovered a significant upregulation of angiogenesis markers, namely vegfa, vegfb, and FGF2, implying that PSAT1 triggers angiogenesis post-MI (Figure 3L). In *in vitro* cultures of NRVMs and neonatal rat cardiac fibroblast, we found that the PSAT1 modRNA mostly induces above angiogenesis markers in NRVMs, but not in the fibroblasts (Figure S5A-C). As a result of improved cardiac function, reduced scar size, induced CM proliferation, and angiogenesis, survival of PSAT1-injected mice was also significantly improved (Figure 3M). Taken together, our data confirm that PSAT1 modRNA expression promotes cardiomyocyte proliferation, enhances cardiac function, and stimulates angiogenesis, while also reducing the scar size, suggesting a beneficial cardiac remodeling post-MI.

### Serine synthesis pathway regulates CM proliferation and apoptosis

**Figure 4:**
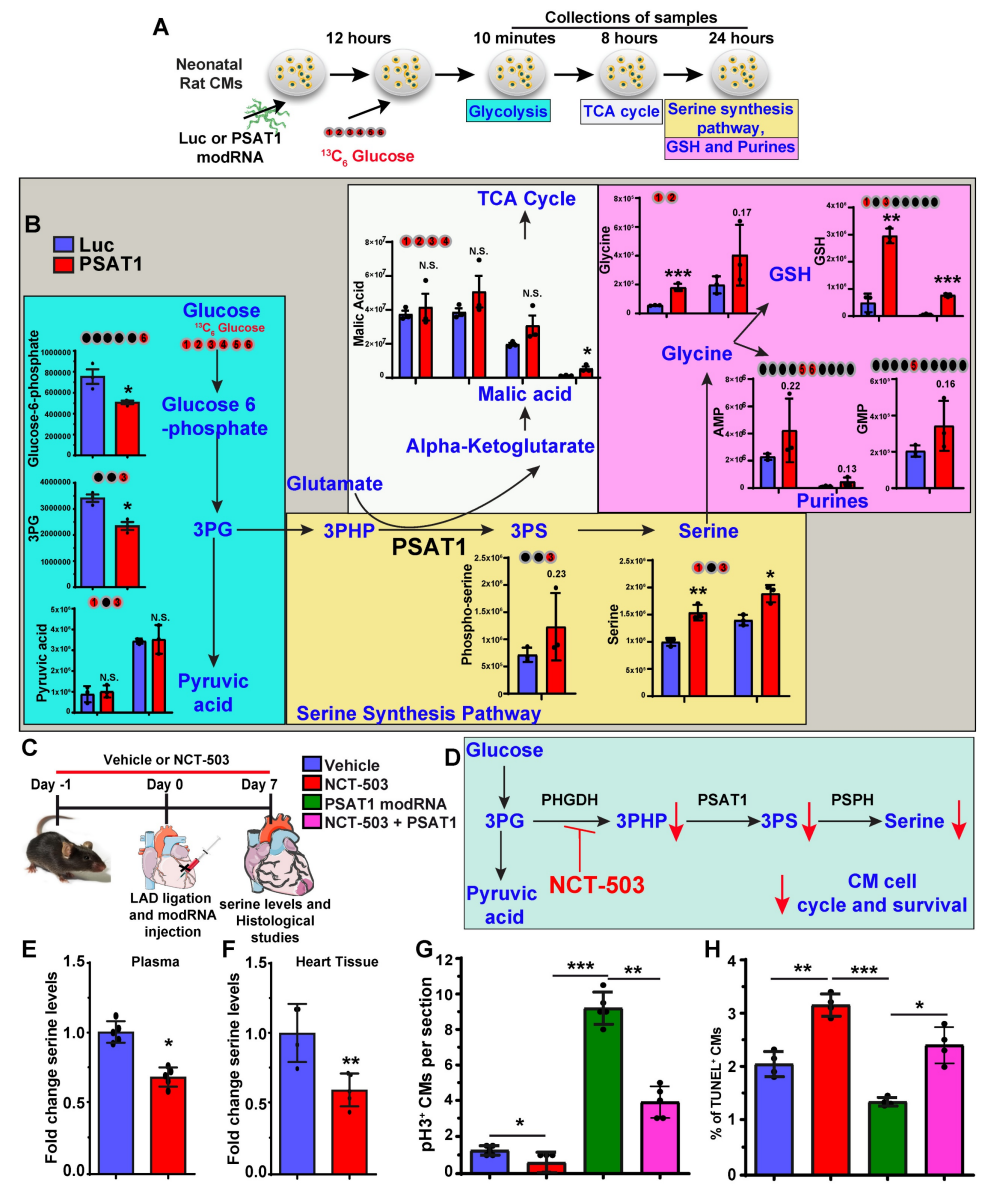
** Serine synthesis pathway regulates CM proliferation and apoptosis. A.** Experimental timeline and analysis of absolute intracellular labeled metabolites with [U-^13^C_6_] glucose flux using mass spectrometry of lysates from P2-P3 NRVMs transfected with PSAT1 or Luc modRNA. **B.** The levels of [U-^13^C]-labeled glycolysis metabolites were evaluated 10 mins after [U-^13^C_6_] glucose addition, TCA metabolites were evaluated 8 h. after [U-^13^C_6_] glucose addition and serine and nucleotides synthesis pathway metabolites were evaluated 24 h after [U-^13^C_6_] glucose addition (n = 3). In all panels, the X-axis represents the carbon number labelled with [U-^13^C_6_] in the given structure of a specific molecule (shown as red ball with number) and the Y-axis represents absolute intensity. **C**. Experimental plan for inhibition of serine synthesis pathway by PHGDH inhibitor, NCT-503 in presence or absence of PSAT1 modRNA in mice model of MI. **D.** Proposed metabolic mechanism by which NCT-503 treatment impedes CM cell cycle and survival by inhibiting SSP. **E-F.** Quantitative analysis of serine levels in plasma and heart tissue 7 days post-MI showing inhibition of SSP (n = 4-6).** G**. Quantitative analysis of CM cell cycle by pH3^+^ CMs 7 days post-MI after treatment with or without and NCT-503 and PSAT1 modRNA (n = 4-6). **H.** Quantitative analysis of TUNEL^+^ CMs 7 days post-MI (n = 4-6). Unpaired two-tailed t-test B, E and F. One-way ANOVA for G-H. ***, P < 0.001, **, P < 0.01.

PSAT1 is an enzyme involved in the serine synthesis pathway, where it converts 3-phosphohydroxy-pyruvate (3PHP), a SSP intermediate, and glutamate into phosphoserine (3PS) and α-ketoglutarate which enters the TCA cycle. The 3PS intermediate is further enzymatically converted into serine. To analyze whether serine synthesis and other metabolites of the SSP and/or downstream metabolic pathways increase under PSAT1 modRNA overexpression, metabolic flux analyses were performed by using isotopic tracer ^13^C_6_ glucose-supplemented media in NRVMs. NRVMs were cultured following isolation and then treated with modRNA. Media was supplemented with ^13^C_6_ glucose 12 h post-delivery of PSAT1 or Luc modRNA for analysis of glycolysis (10 mins after addition of ^13^C_6_ glucose), TCA cycle (8 h) or SSP (24 h) (Figure 4A-B). Our results show a significant elevation of the metabolites involved in the SSP, including phosphoserine, serine, and glycine, suggesting upregulation of the serine biosynthesis pathway (Figure 4B). Furthermore, the high glycine levels are precursors for glutathione (or GSH, which inhibits ROS production) and purine nucleotide synthesis (60. We also observed PSAT1 induced regulation of metabolites involved in glycolysis and TCA cycle (Figure 4B, and Figure S6A-B). Similarly, the PSAT1 modRNA injection post-MI resulted in an increase in the levels of serine, glutamic acid and glycine (HPLC study), which correlate with our NRVM ^13^C_6_ glucose flux studies Figure S7A-E). We observed increased glutathione and both purine (AMP and GMP) levels after PSAT1 modRNA expression suggesting these increased purines likely provide the raw material for DNA synthesis in post-natal CMs (Figure 4B). Next, we analyzed whether inhibition of SSP has any effect on CM proliferation and apoptosis post-MI (Figure 4C). We used PHGDH (first rate-limiting enzyme in SSP) inhibitor, NCT-503 (30 mg/kg for each mouse), delivered intraperitoneally once daily *in vivo* (Figure 4C-D). NCT-503 inhibits serine levels in plasma and heart tissues, analyzed 7 days post-MI (Figure 4E-F). The treatment of NCT-503 significantly impedes the CM cell cycle (pH3^+^ CMs) at the basal level 7 days post-MI (Figure 4G). At the same time, PSAT1 modRNA expression under SSP inhibition by NCT-503 partially reversed the CM mitosis compared to the control, but at a significantly lower rate than PSAT1 modRNA alone (Figure 4G). Furthermore, NCT-503 treatment significantly increased the CM apoptosis (analyzed by TUNEL^+^ CMs) 7 days post-MI. While PSAT1 modRNA alone significantly inhibited CM apoptosis, NCT-503 treatment inhibits the PSAT1-induced anti-apoptotic effect in CMs (Figure 4H). In sum, our data suggest PSAT1 induces SSP in CMs, increases GSH, and provides nucleotides for DNA synthesis. Increased GSH level hinders oxidative stress and inhibits CM apoptosis. In contrast, inhibition of SSP by small molecule like NCT-503 promotes CM apoptosis and impedes CM proliferation; establishing for the first time that SSP plays a key role in regulating CM proliferation and apoptosis post-MI.

### PSAT1 modRNA inhibits CM apoptosis, oxidative stress, and DNA damage post-MI

**Figure 5:**
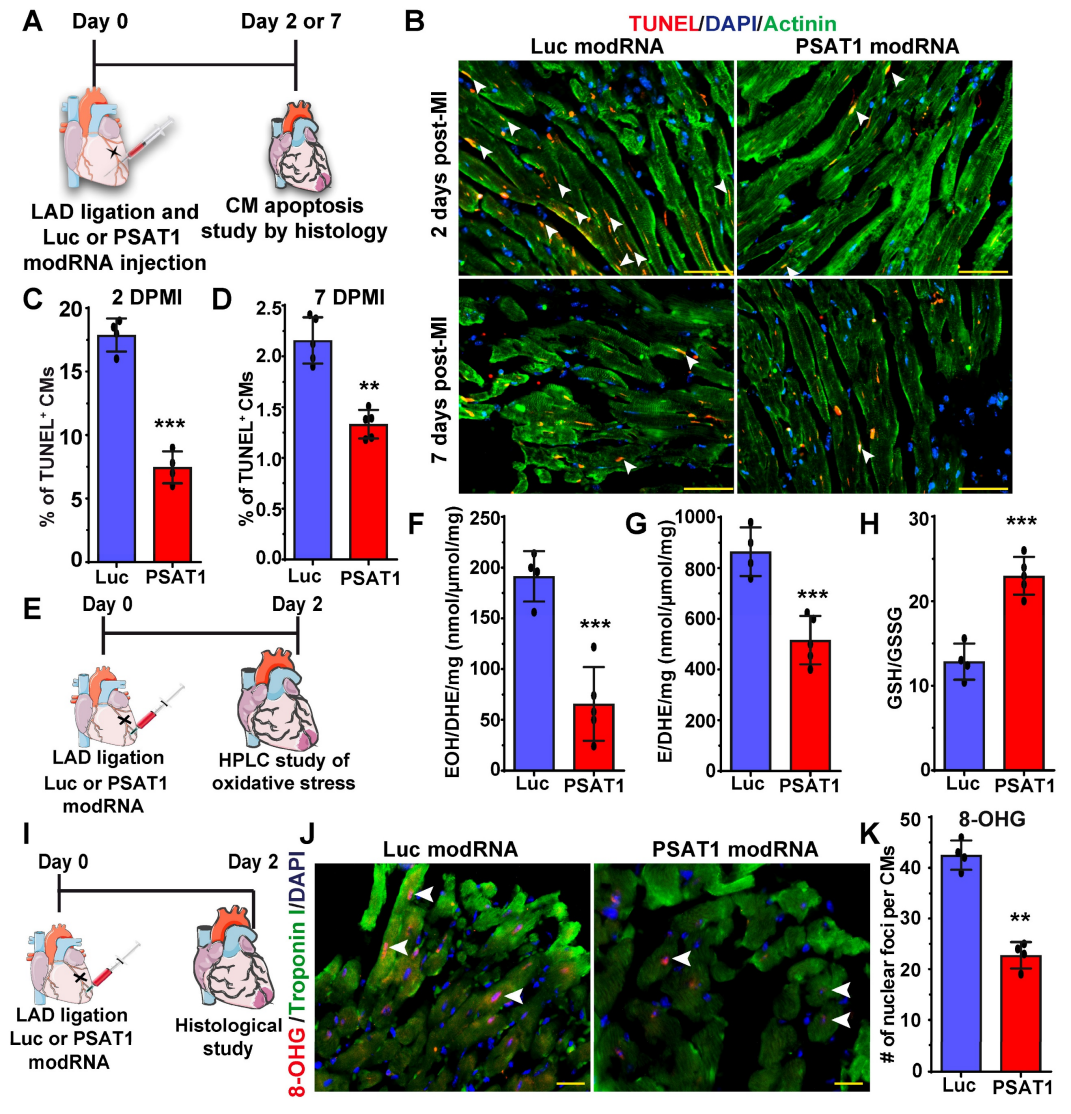
** PSAT1 modRNA inhibits CM apoptosis and oxidative stress post-MI. A.** Experimental timeline used for evaluating the effect of PSAT1 or Luc modRNA delivery on CM apoptosis in a mouse MI model using the TUNEL method. **B**. Representative images of TUNEL staining 2- or 7-days post-transfection of Luc or PSAT1 modRNA injection and MI, Red (TUNEL^+^), Green-CM-specific marker (α-sarcomeric actinin^+^) and DAPI-nucleus marker (arrow showing TUNEL^+^ CM). **C**-**D**. Quantitative analysis of TUNEL^+^ CMs from B, 2 days (C) or 7 days (D) post-MI and Luc or PSAT1 modRNA injection showing reduction in CM apoptosis (n = 5). **E**, Experimental timeline used to evaluate reactive oxygen species (ROS) levels after MI and delivery of Luc or PSAT1 modRNA. **F-G**. HPLC quantification of ROS, superoxide probe dihydroethidium (DHE) to 2-hydroxyethidium (EOH) in F, or ethidium (E) in G, (n = 4 or 5), 2 days after MI and PSAT1 or Luc modRNA injection. **H.** Quantification of GSH/GSSG ratio (reduced/oxidized glutathione), (n = 4 or 5) 2 days post-MI and modRNA injection indicating significant decrease in oxidative stress. **I.** An experimental timeline was used to evaluate the effect of PSAT1 or Luc modRNA delivery on DNA damage in CMs in a mouse MI model.** J.** Representative images of 8-OHG (8-hyrdroxyguanosine) foci frequency in CMs 2 days post-MI and transfection with either PSAT1 or Luc modRNA (co-stained for troponin I and DAPI). **K**. Quantitative analysis of J, (n = 4). Unpaired two-tailed t-test C-D, F-H, and K ***, P < 0.001, **, P < 0.01. Scale bar = 75 μm (B), 25 μm (J).

Millions of CMs die after MI due to oxidative stress; therefore, to confirm our in vitro studies and investigate whether PSAT1 can inhibit CM apoptosis in vivo, we used a mouse model of MI and delivered PSAT1 modRNA (Figure 5A). We found that PSAT1 significantly decreased the number of TUNEL^+^ CMs at 2 and 7 days, post-MI at border zone and infarct area in LV (Figure 5B-D). We also analyzed the expression of apoptosis marker genes and observed that hearts injected with PSAT1 modRNA exhibited significantly increased expression of antiapoptotic genes like BCL2 and Bcl-xL, accompanied by a downregulation of pro-apoptotic genes like Bak1, Bax, and Caspase 3 levels compared to hearts injected with Luc modRNA (Figure S8A-B). Increased oxidative stress and DNA damage in mouse heart induces CM apoptosis and inhibits CM cell cycle (61, 62. To analyze the effect of PSAT1 on oxidative stress post-MI, we performed HPLC measurements of oxidative stress (GSH/GSSG ratio) superoxide and other ROS. HPLC detection of a superoxide probe dihydroethidium (DHE) shown a significant decrease in both 2-hydroxyethidium (EOH), a particular product for superoxide anion radical, and in ethidium (E), oxidized by other reactive oxygen species such as H2O2 (Figure 5E-H). The data also showed PSAT1 significantly increased the GSH/GSSG ratio (Figure 5H). To investigate whether PSAT1 modRNA expression has any role on the levels of reducing agents (NADPH and NADH) in the heart, we analyzed their levels by HPLC. We found there is a trend towards higher levels of reducing agents like NADPH and NADH after myocardial delivery of PSAT1 modRNA post-MI Figure S9A-C). To study DNA damage post-MI and modRNA injection, we used the 8-OHG (8-oxo-7,8-dihydroguanine [8-oxoG) to quantify oxidative base modification of DNA and DNA damage. Immunostaining analysis of heart sections showed significantly fewer positive foci for the nuclear 8-OHG (nuclear foci per CMs) two days post-MI after myocardial delivery of PSAT1 modRNA compared to Luc modRNA (Figure 5I-K). We here show that inhibition of ROS and ROS-mediated activation of DNA damage using the PSAT1 modRNA can induce CM proliferation and significantly inhibit CM apoptosis post-MI.

### YAP1-PSAT1-β-catenin molecular axis induces CM proliferation and cardiac repair

**Figure 6:**
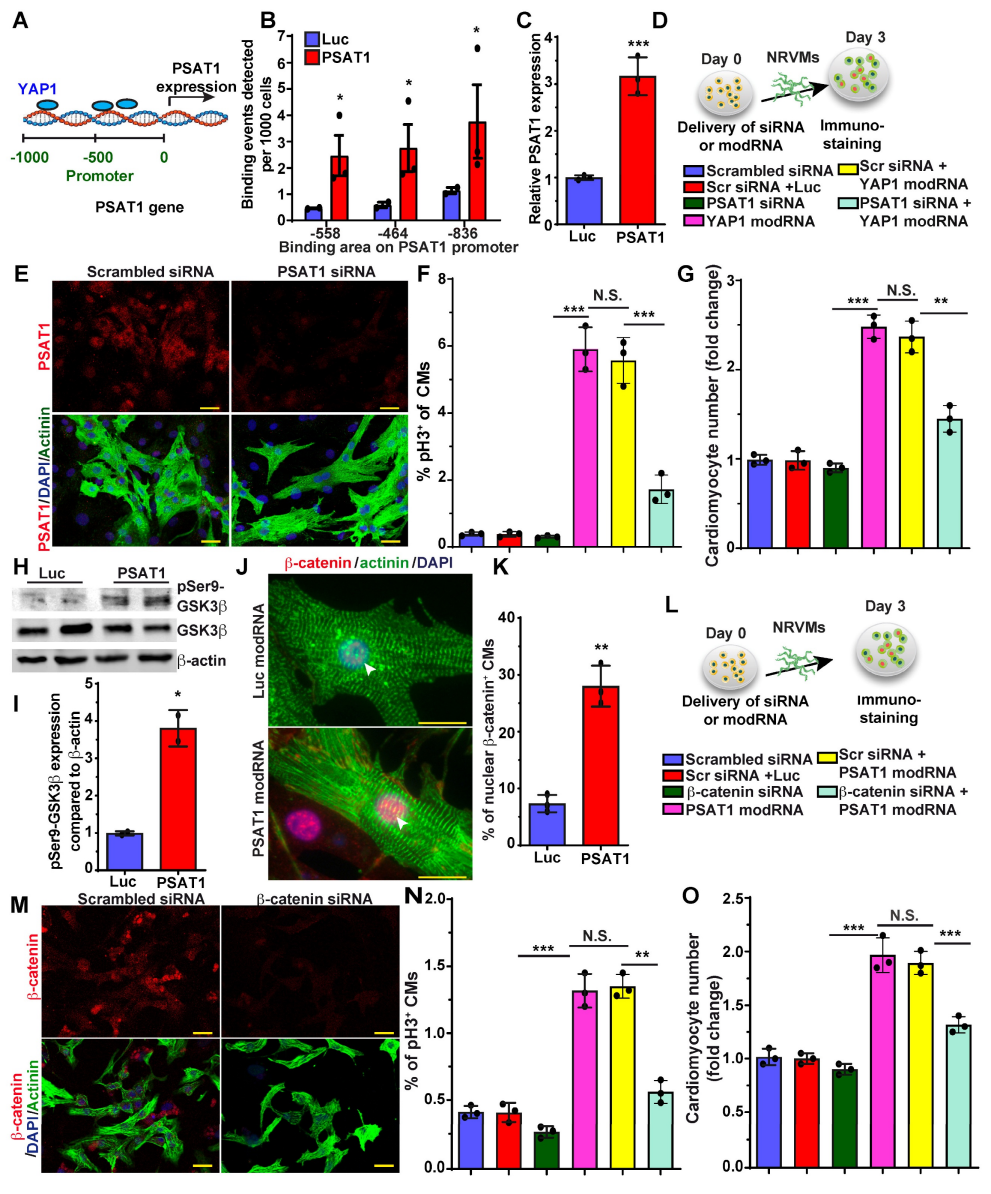
** YAP1-PSAT1-β-catenin molecular axis induces CM cell cycle. A.** Representative diagram showing YAP1 binds (ChiP-qPCR analysis) to the PSAT1 promoter sites in NRVMs. **B.** Quantitative analysis of YAP binding to the promoter region of PSAT1 as shown in A, with negative control primers, confirming YAP1 is trans-activator of PSAT1 (n = 3). **C.** Quantitative analysis showing PSAT1 mRNA levels after Luc or YAP1 modRNA expression in NRVMs. (n = 3). **D.** Experimental timeline to study the effect of PSAT1 inhibition (by siRNA) on YAP1 induced CM cell cycle in NRVMs (Knock-down of PSAT1 inhibits YAP1 modRNA-induced CM cell cycle). Cells were fixed after 3 days post-transfection (respective siRNA or and modRNA) and immunostained with antibodies to pH3 or CM marker α-sarcomeric actinin. **E.** Representative images of PSAT1 inhibition 3 days post-transfection with scrambled or PSAT1 siRNA, red (PSAT1^+^), green (α-sarcomeric actinin^+^) and DAPI-nucleus marker. **F.** Quantitative analysis of pH3^+^ (D) CMs 3 days post-transfection (n = 3). **G.** Quantitative analysis of CM numbers (n = 3). This data suggest inhibition of PSAT1 dampens the YAP1 induced CM proliferation. **H.** Western-blot analysis of PSAT1-induced phosphorylation of Ser9 of GSK-3β in NRVMs compared to Luc modRNA. **I.** Quantitative analysis of differential protein expression in G (n = 2). **J.** Representative images of β-catenin in CMs nucleus and cytoplasm 48 h. after PSAT1 modRNA (co-stained α-sarcomeric actinin and DAPI). **K.** Quantitative analysis nuclear β-catenin^+^ CMs, Nuclear transfer of β-catenin in CMs suggests activation and stabilization of β-catenin post-PSAT1 modRNA transfection (n = 5).** L**. Experimental timeline to study the effect of β-catenin inhibition (by siRNA) on PSAT1 induced CM cell cycle on NRVMs. Cells were fixed after 3 days post-transfection (respective siRNA or/and modRNA) and immunostained for pH3 antibody and CM marker α-sarcomeric actinin. M. Representative images of β-catenin inhibition 3 days post-transfection of scrambled or β-catenin siRNA transfection, Red (β-catenin^+^), Green-CM-specific marker (α-sarcomeric actinin^+^) and DAPI-nucleus marker. **N.** Quantitative analysis of pH3^+^ (M) CMs 3 days post-transfection (n = 3).** O.** Quantitative analysis of CMs number (n = 3). This data suggests inhibition of β-catenin suppress the PSAT1 induced CM proliferation. Unpaired two-tailed t-test for B-C, I-K, One-way ANOVA for F-G, N-O. ***, P < 0.001, **, P < 0.01*, P < 0.05. Scale bar = 25 μm (E and J), 50 μm (M).

YAP1 is a master regulator of CM proliferation and cardiac repair in post-MI and in heart failure mice models (30, 31, 63, 64. There is much data on YAP1 upstream regulators in CM proliferation and cardiac repair, but knowledge of its downstream molecular signaling pathway is limited and warrants further study in CMs. Recently it was shown that YAP1 induces expression of PSAT1 mRNA in tumors 65. To analyze the role of YAP1 in PSAT1 expression in CMs, we treated NRVMs with YAP1 modRNA and found that YAP1 modRNA significantly upregulated PSAT1 mRNA expression (Figure 6C). To determine whether YAP1 directly binds to the promoter region of PSAT1 to increase its transcription or if YAP1 regulates PSAT1 indirectly, we treated NRVMs with YAP1 modRNA and performed ChIP-qPCR (Figure 6A-B). We found that anti-YAP1 antibodies-pulldown PSAT1 genomic DNA fragment that spanned the region encompassing three putative YAP1 binding sites at -836bp, -558bp, and -464bp of PSAT1 promoter) suggesting that YAP1 induces PSAT1 mRNA expression in CMs (Figure 6B-C). To see whether PSAT1 inhibition affects the YAP1-induced CM cell cycle, we treated NRVMs with scrambled siRNA or PSAT1 siRNA alone or in the presence of YAP1 modRNA (Figure 6D). We found that PSAT1 siRNA significantly inhibits the CM cell cycle (pH3+ CMs) and reduces CM numbers in the presence of YAP1 modRNA, showing that YAP1-induced CM cell cycle is specifically inhibited by knockdown of PSAT1, supporting that YAP1 requires PSAT1 to exert its effect on CM proliferation (Figure 6D-G).

Nuclear β-catenin interacts with YAP1 to induce CM proliferation and cardiac regeneration 30. Yet, how inhibition of the hippo pathway or how activation of YAP1 brings β-catenin to the nucleus is not well studied in CMs. Therefore, we investigated the role of PSAT1 modRNA expression on the Wnt pathway, specifically focusing on β-catenin. We found that PSAT1 modRNA expression in NRVM induces phosphorylation of GSK3β at Ser9, an upstream regulator of β-catenin (Figure 6H-I).

The increased Ser9P-GSK3β stabilizes β-catenin in the cytoplasm and prevents proteolytic degradation, as shown before 66, 67. We assessed by immunostaining whether the stabilized β-catenin can translocate to the nucleus. The introduction of PSAT1 modRNA yielded clearly detectable or increased β-catenin in CM nuclei, implying that PSAT1 stabilized β-catenin and facilitated nuclear translocation *in vitro* and post-MI (Figure 6J-K) Figure S10A-B). To study whether β-catenin inhibition alters the PSAT1-induced CM cell cycle, we treated NRVMs with scrambled siRNA or β-catenin siRNA alone or in the presence of PSAT1 modRNA. β-catenin inhibition significantly inhibited the CM cell cycle (pH3^+^ CMs) and reduced CM numbers in the presence of PSAT1 modRNA, suggesting involvement of β-catenin in PSAT1-induced CM cell cycle (Figure 6M-O). Taken together, our data indicates that YAP1 binds to the promoter of PSAT1 (YAP1 transactivates PSAT1) and induces its expression. PSAT1 modRNA delivery activates CM cell cycle by modulating cell cycle genes and by stabilizing β-catenin through GSK3β inhibition leading to nuclear translocation of β-catenin to interact with available YAP1 as shown earlier (30.

**Figure 7:**
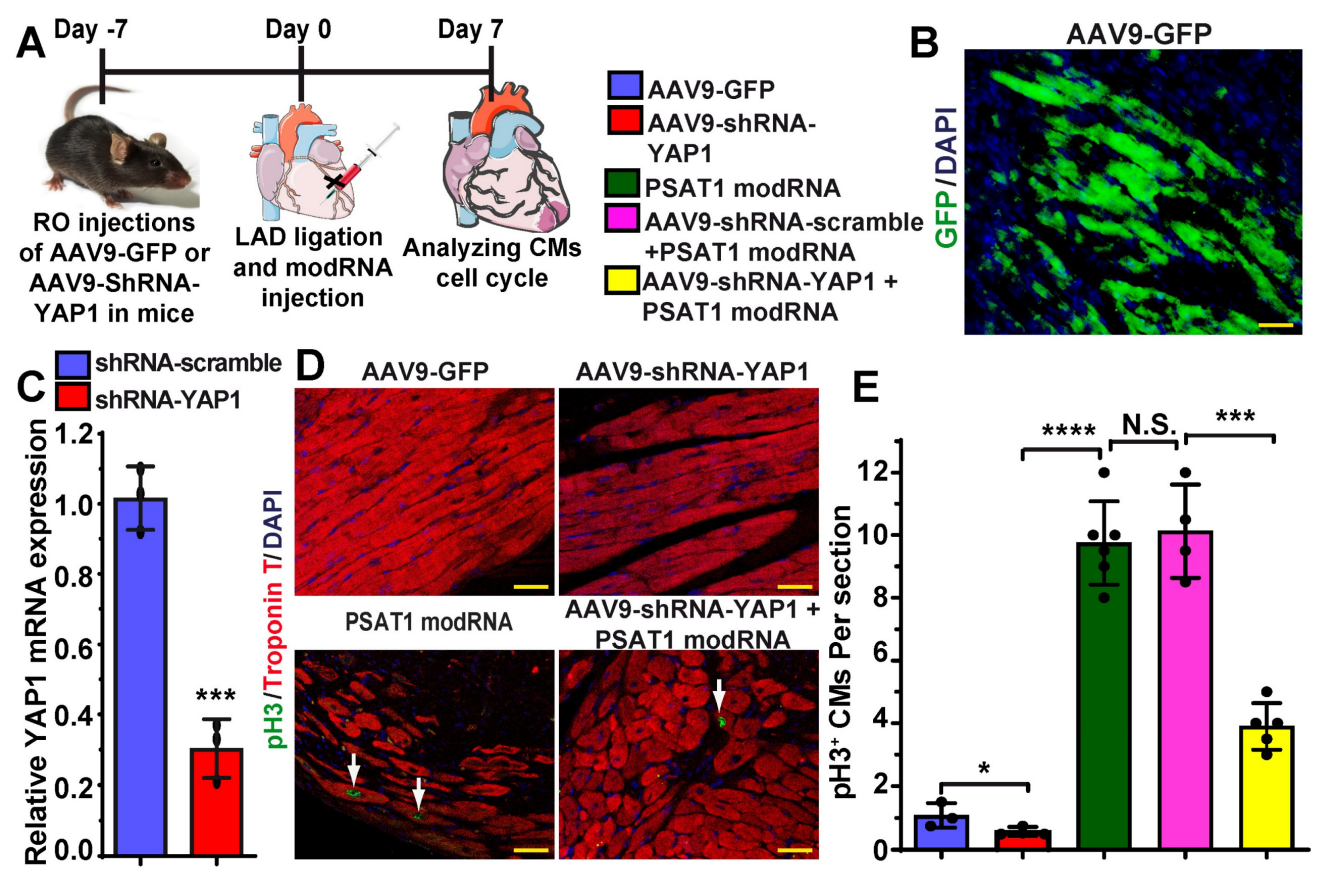
** YAP1 inhibition diminishes PSAT1-induced CM cell cycle post-MI. A.** To study the effect of YAP1 inhibition on PSAT1-induced CM cell cycle in the heart, AAV9-GFP or AAV9-YAP1-shRNA was retro-orbitally injected in mice 7 days before LAD ligation and PSAT1 modRNA delivery. Hearts were harvested 7 days post-MI for immunostaining. **B.** Representative images of GFP^+^ (green) cells and DAPI (blue) 7 days post-MI after delivering AAV9-GFP. **C.** Quantitative analysis showing YAP1 mRNA expression in mouse heart post-delivery of scramble or YAP1 shRNA (n = 3). **D.** Representative images of pH3^+^ (green) CMs, CM-specific marker Troponin T^+^ (red) and DAPI (blue) 7 days post-MI. **E.** Quantitative analysis of pH3^+^ CMs in C, showing inhibition of YAP1 in heart dampened the PSAT1 induced CM mitosis post-MI (n = 3-6). One-way ANOVA for E, Unpaired two-tailed t-test for C. ****, P < 0.0001, **, P < 0.01, *, P < 0.05, Scale bar = 100 μm (B and D).

**Figure 8:**
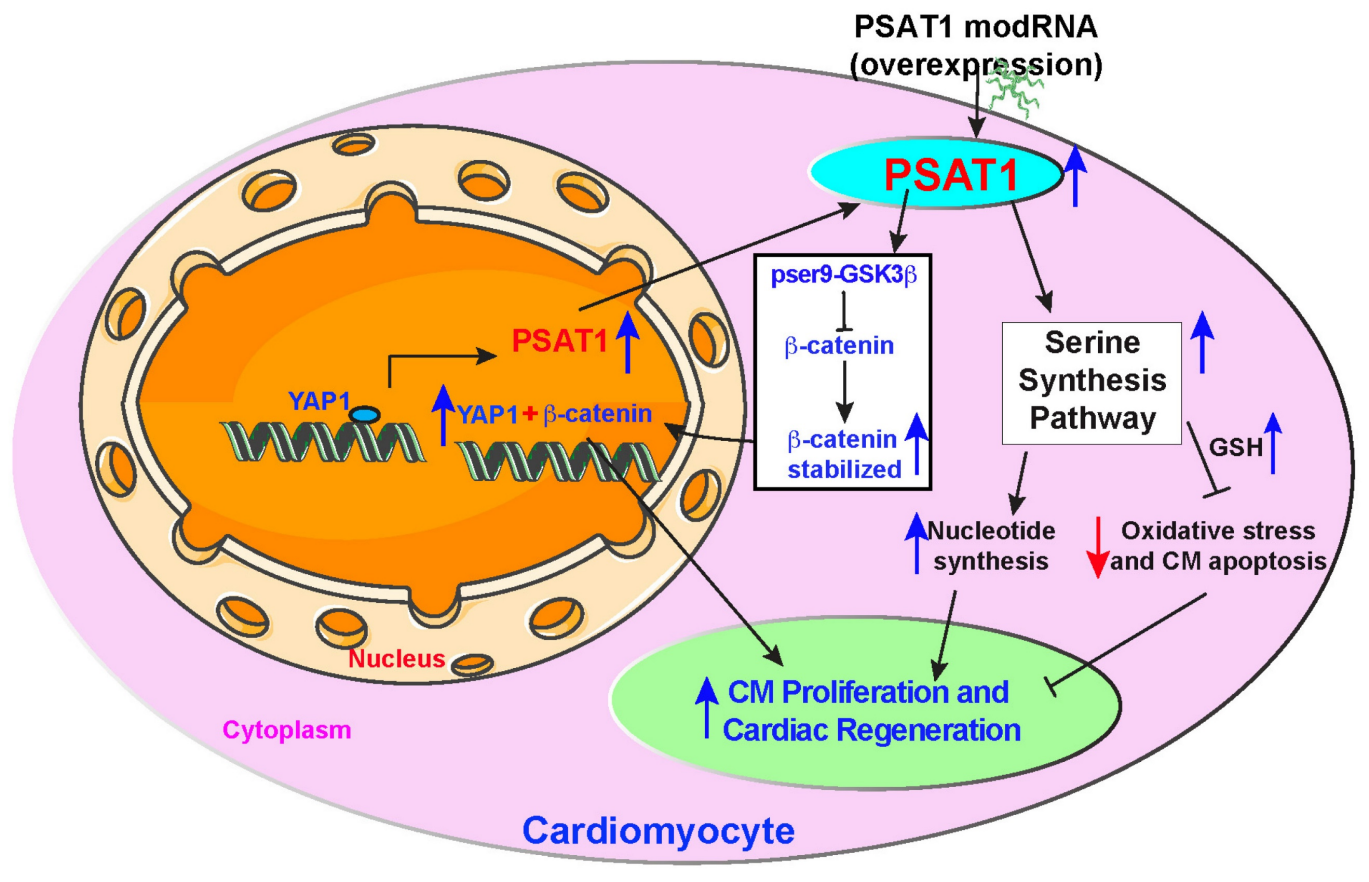
** Proposed PSAT1 induced molecular and metabolic signaling pathway in cardiac repair.** PSAT1 induces CM proliferation and cardiac repair in the heart by two different pathways. At the molecular level, PSAT1 induces the YAP1-β-catenin molecular axis, and metabolically it activates the SSP. YAP1 transactivates PSAT1, PSAT1, in turn, stabilizes β-catenin by phosphorylating Ser9 of GSK3β, and β-catenin translocate to the nucleus and interacts with YAP1, resulting in CM proliferation and cardiac repair. SSP induces CM proliferation by activating the nucleotide synthesis pathway and inhibiting oxidative stress, CM apoptosis, and DNA damage response by generating higher glutathione (GSH) levels.

To investigate whether PSAT1 is upstream of YAP1-β-catenin nuclear interaction and whether YAP1 inhibition affects PSAT1-induced CM cell cycle in the heart post-MI, we retro-orbitally injected AAV9-GFP, AAV9-shRNA-scramble or AAV9-shRNA-YAP1 seven days before LAD ligation and PSAT1 modRNA delivery to infarcted myocardium (Figure 7A-E). Seven days after LAD and modRNA injection, we found that AAV9-shRNA-YAP1 significantly inhibited YAP1 expression in the heart and reduced CM cell cycle (pH3^+^ CMs) to basal level, while PSAT1 modRNA expression under YAP1 inhibition partially induced CM cell cycle compared to PSAT1 modRNA alone or in presence of AAV9-shRNA-scramble (Figure 7C-E). These data indicate that PSAT1 is upstream of the YAP1-β-catenin interaction/complex in the nucleus. Knockdown of YAP1 inhibits PSAT1-induced CM cell cycle suggesting the importance of the YAP1-PSAT1 molecular axis and implicates YAP1-β-catenin interaction in the nucleus as the mechanism by which PSAT1 regulates CM proliferation (Figure 8).

## Discussion

In summary, our study identifies PSAT1 as a novel protein expressed early in mouse heart development and virtually undetectable in healthy adult mouse heart. While PSAT1 overexpression induces CM proliferation and cardiac repair, its expression in CMs in the physiological condition is regulated transcriptionally by a master regulator of cardiac repair, YAP1. Single myocardial delivery of PSAT1 modRNA induces CM proliferation, cardiac repair, and angiogenesis, while inhibiting CM apoptosis, oxidative stress, cardiac fibrosis, DNA damage, and yielding functional improvement post-MI by inducing serine and nucleotide synthesis and β-catenin translocation to the nucleus (Figure 8). Our data suggest that PSAT1 modRNA induces multiple processes (pleiotropic effects) to promote favorable cardiac remodeling post-MI. It was shown that homozygous mutation in PSAT1 causes death before weaning in mice, and mutations also result in Neu-Laxova syndrome and phosphoserine aminotransferase deficiency 68-70. Maintaining PSAT1 levels was essential for mESC self-renewal and pluripotency 71. In cancer cell proliferation, PSAT1 regulates cell proliferation through the Wnt/catenin pathway and PSAT1 is regulated by ATF4 72, 73. We propose that PSAT1 is downregulated after birth as cardiomyocytes transition from a proliferative fetal state to a terminally differentiated, non-proliferative postnatal phenotype. This developmental shift may be governed by a combination of mechanisms, including a metabolic switch from glycolysis to oxidative phosphorylation, epigenetic silencing via promoter methylation and repressive histone modifications, transcriptional repression by factors such as p53 and the Rb-E2F pathway, and microRNA-mediated regulation by miR-1, miR-133, and miR-195. Together, these processes enforce cell cycle exit while enhancing contractile function and metabolic efficiency in mature CMs, underscoring PSAT1's intriguing role in cardiac regeneration and its potential as a therapeutic target.

In the mammalian heart, a metabolic switch occurs in the first week after birth when the heart utilizes more fatty acid oxidation than glycolysis as an energy source 61. Increased oxygenation and ROS generation in the postnatal heart inhibits cardiac regeneration in neonatal mice 61. Similarly, substantial metabolic shifts occur in response to abnormal heart conditions like ischemia, hypertrophy, and pressure overload towards glycolytic metabolism from fatty acid oxidation. This shift protects against damage and induces expression of a fetal gene program, including genes involved in glycolytic metabolism 61, 74. Catabolic reactions like lipid oxidation and oxidative phosphorylation sustain energy production for homeostasis in highly energetic organs like the heart. While dividing, cells need to acquire an adequate biomass to support the production of new cells through anabolic pathways including synthesis of new proteins, lipids, carbohydrates, DNA, and RNA molecules. Glucose and glutamine are key in sustaining active metabolic pathways like glycolysis and anaplerotic flux of the TCA cycle in mammalian cells 75. In cells, serine is biosynthesized through a three-step enzymatic reaction. First, 3-phosphoglycerate derived from glycolysis is oxidized into phosphor-hydroxy pyruvate (pPYR) by phosphoglycerate dehydrogenase (PHGDH). Sequentially, pPYR is catalyzed by phosphoserine aminotransferase (PSAT1) to generate phosphoserine, which is then dephosphorylated by 1-3- phosphoserine phosphatase (PSPH) to form serine. External serine and serine derived from glycolysis can be converted to glycine by hydroxy methyl transferase (SHMT), which activates one-carbon metabolism 75. Both serine and glycine provide essential precursors for synthesizing proteins, nucleic acids (purines), and lipids crucial to homeostasis 75. Glycine is a precursor for glutathione (GSH), a potent antioxidant that protects cells from oxidative stress by neutralizing ROS. The inhibition of ROS or oxidative stress is a significant element in the regenerative response of mouse hearts during development and in adult mice after heart injury 61. Our study shows that PSAT1 modRNA-induced anabolic shift is conductively coupled CM proliferation. De novo serine biosynthesis from glycerate-3P is integral to this shift and is needed for CM proliferation in development or in response to injury.

Our ^13^C_6_-glucose isotopic tracers for metabolic flux analysis showed PSAT1 modRNA significantly induced SSP and glycine levels in CMs, which results in increased levels of GSH, suggesting a more reducing environment. ROS can cause cellular oxidative stress and results in damage to proteins, lipids, and nucleic acids 74. The ROS-mediated activation of oxidative stress and DNA damage is a critical upstream event that mediates cell-cycle arrest not only during mouse heart development but also induces deleterious cardiac remodeling in ischemic heart diseases. Inhibition of oxidative stress, or ROS, will help to induce the CM cell cycle or inhibit CM apoptosis 61, 62. As a result of SSP activation through PSAT1 modRNA, we have seen reduced ROS levels (HPLC analysis), increased GSH to GSSG ratio in mice hearts post-MI, and significantly increased GSH levels in vitro in CMs. The reduced-to-oxidized glutathione ratio is broadly used as an indicator of oxidative stress. Oxidative stress or ROS induction after MI results in the widespread activation of DNA damage and DNA damage responses. In the analysis of 8-OHG, a marker of DNA damage, was significantly reduced in CMs 61. Increased DNA damage induces senescence and cell death 76. As a result of activated SSP, increased glycine and GSH levels and decreased DNA damage facilitated by PSAT1 modRNA administration result in a significant reduction in CM apoptosis at 2- and 7-days post-MI. Activation of SSP and glycine levels also induces the synthesis of purine nucleotides. The nucleotides are a prerequisite for the cell to enter S-phase of the cell cycle. The increased nucleotides after PSAT1 modRNA delivery allowed CMs to enter cell cycle and use available nucleotides for the s-phase. Our data link the SSP to cardiac repair providing direct evidence that PSAT1-induced enhanced SSP is required for metabolic reprogramming of CMs, generating the metabolites for anabolic pathways, and providing reducing agents to reverse oxidative stress to promote CMs to undergo cell cycle and myocardial repair (Figure 8).

Inhibition of the hippo pathway induces CM proliferation and cardiac regeneration by activating the Wnt/β-catenin signaling pathway and the interaction of YAP1 and β-catenin in the nucleus 30, 31, 63, 64, 77, 78. Yet it is unknown how hippo inhibition or YAP1 overexpression in heart or CMs bring β-catenin into the nucleus. First, we showed by ChiP-qPCR analysis that YAP1 is a trans-activator of PSAT1. Second, PSAT1 modRNA induces stabilization of β-catenin and its translocation to the CM nucleus by phosphorylation of Ser9 of GSK-3β, an upstream regulator of β-catenin. This results in the interaction of YAP1 and β-catenin in the nucleus, which is a prerequisite for YAP1-induced CM proliferation. To study whether PSAT1 is upstream of YAP1-β-catenin interaction and plays a vital role in regulating YAP1-β-catenin interaction in the nucleus and CM cell cycle, we inhibited YAP1 or β-catenin using AAV-ShRNA YAP1 or β-catenin siRNA, respectively, in CMs. We found YAP1 or β-catenin inhibition in CMs markedly suppresses the PSAT1-induced CM cell cycle post-MI and in cultured NRVMs. These data suggest that PSAT1 regulates YAP1-β-catenin function in the nucleus by bringing β-catenin into the nucleus, resulting in its interaction with YAP1 to activate a CM proliferation program. Importantly, our data suggest that at the molecular level, PSAT1 is a molecular link in the YAP1-β-catenin axis, inducing robust CM proliferation and cardiac repair post-MI. The precise crosstalk between PSAT1's metabolic function and its regulation of signaling pathways remains to be fully elucidated, we speculate that metabolites generated through the SSP may serve as upstream modulators or co-factors that fine-tune the activity of these signaling cascades. For example, enhanced NADPH production may stabilize β-catenin indirectly via redox-sensitive regulation of GSK3β activity, while increased serine flux could influence epigenetic or transcriptional changes that support YAP-mediated gene expression. Future studies utilizing catalytically inactive PSAT1 mutants or metabolic flux analysis to dissect the enzyme-dependent versus signaling-dependent contributions of PSAT1 in myocardial repair.

Furthermore, we observed that PSAT1 modRNA treatment enhances endothelial cell proliferation and increases the expression of key pro-angiogenic factors, including VEGF-A, VEGF-B, and FGF2 in the heart, suggesting a strong pro-angiogenic effect. Although the precise molecular mechanisms remain to be fully elucidated, current evidence indicates that PSAT1, a critical enzyme in the serine biosynthesis pathway, may promote angiogenesis by modulating cellular metabolism—particularly nucleotide synthesis and redox balance, which are vital for endothelial cell function and proliferation. Additionally, PSAT1 may influence angiogenesis through activation of YAP/β-catenin signaling, a pathway also known to regulate vascular growth. These findings highlight the need for further investigation to delineate the precise mechanisms by which PSAT1 regulates angiogenesis.

The clinical potential of PSAT1 modRNA for ischemic heart diseases is promising, offering benefits such as promoting cardiomyocyte proliferation, inhibiting oxidative stress and cell death, enhancing cardiac repair, and improving post-MI function. PSAT1 modRNA leverages SSP modulation and YAP1-β-catenin signaling to drive survival, proliferation, and regeneration, but its feasibility and safety in humans require thorough evaluation. In our study, we utilized mouse models to establish the foundational efficacy (PSAT1 mRNA expression, cardiac repair, and functional improvement) and safety of our therapeutic approach under physiologically relevant conditions. To bridge the translational gap, we deliberately focused on highly conserved molecular pathways, including YAP1-β-catenin signaling and the serine synthesis pathway (metabolism), which are implicated in both mouse and human cardiovascular pathophysiology. This cross-species conservation provides a strong basis for hypothesizing similar therapeutic benefits in human systems. Additionally, PSAT1 amino acid sequence shows approximately 95% conservation between mice and humans, with the mRNA sequence about 85-90% conserved, underscoring the evolutionary preservation of its function. This high degree of conservation supports the hypothesis that PSAT1 could have similar therapeutic benefits in humans, given its critical role in serine metabolism and other cellular processes. Although the foundational studies were conducted in rodent models, progression to large animal models is a necessary step before any potential human application due to physiological similarities that more closely resemble humans.

The direct intramyocardial injection approach used in our study holds strong clinical relevance, particularly in the context of coronary artery bypass grafting (CABG). During CABG procedures, cardiac surgeons have direct access to the myocardium, providing an opportunity for targeted, localized delivery of mRNA, without the need for additional invasive procedures. This makes intramyocardial injection of mRNA a feasible and potentially translatable delivery route for patients with ischemic heart disease. To enhance the clinical applicability of PSAT1 modRNA, we are considering advanced delivery platforms such as lipid nanoparticles (LNPs) or Specific Modified mRNA Translation systems (SMRTs) 51. These technologies are designed to minimize off-target effects and ensure targeted delivery to cardiac tissues. The transient nature of modRNA expression is advantageous as it provides sufficient short-term protein production to trigger tissue repair without the risks associated with prolonged overexpression. The success of modRNA-based therapies, especially evidenced by COVID-19 vaccines, demonstrates the feasibility, scalability, and safety of this approach in a clinical setting. Extensive clinical and preclinical studies have validated that the modRNA platform can achieve robust protein expression and functional recovery with minimal immune response or toxicity. However, moving forward, there are several translational challenges to address. These include optimizing the dosage and frequency to maximize therapeutic benefits while minimizing risks, extending preclinical studies to larger animal models to validate efficacy and safety, and assessing long-term effects such as immune activation and tissue remodeling. Regulatory compliance will also be critical as we progress toward clinical trials.

Overall, the promising short-term data and the rigorous roadmap for translational validation we plan to incorporate in our studies indicate a viable path toward clinical application of PSAT1 mRNA therapy for cardiovascular diseases.

## Conclusions

In summary, our studies demonstrate a novel mechanism by which an amino acid synthesis enzyme (PSAT1) licenses CM proliferation and survival through the activation of multiple processes (pleiotropic effects) and provides a potential strategy for cardiac repair (Figure 8). The extensive study of serine synthesis pathway integration with other metabolic pathways (glycolysis, TCA, PPP, and fatty acid metabolism) and their regulation by master regulators of cardiac repair (PSAT1, YAP1, and β-catenin) are an area for future studies. Our data, coupled with past reports, indicate that the modRNA approach can be used for gene expression in the mouse heart, and that a single administration of specific modRNA is sufficient to illicit a regenerative response in the heart post-MI 10, 47, 48.

## Supplementary Material

Supplementary figures.

## Figures and Tables

**Table 1:** **Open reading frame sequences used for modRNA production for this study.** Here we showed the open reading frames (mRNA sequence) of mice or firefly origins to synthesize the modRNA for this study. The modRNA was synthesizes as shown in methods and materials part.

Gene	Open Reading Frame
PSAT1 (mice)	Atggaagccaccaagcaagtggttaactttgggcccgggcctgccaagctgccacactcggtattgttggagatccagaagcagctactagactacagaggactcggcatcagtgtgctcgaaatgagtcacaggtcatcagattttgccaagattattggcaatacagagaatcttgtgagggaattgctagctgttcccaacaactacaaggtgatctttgtacaaggaggtgggtctggccagttcagtgctgtccccttaaatctgattggcctgaaagctggaaggagtgctgactacgtggtgaccggagcttggtcagctaaggctgcggaagaagccaagaagtttggaacggtgaacattgtccaccctaaacttggaagttacacaaaaattccagacccaagcacctggaacctcaacccggacgcctcctatgtatacttctgtgcaaacgagactgtgcacggggtggagtttgacttcgtacctgacgtcaagggagcggtgctggtctgtgacatgtcctcaaacttcttatccaggccggtggatgtttccaagtttggtgtgattttcgctggtgctcagaagaatgttggctctgccggggtgacggtggtgattgtccgggatgacctgctggggttctcgctcagagagtgcccatcagtccttgactacaaagtgcaggctgggaacaactctttgtataacacacctccgtgcttcagcatctacgtcatgggcatggtcctggaatggatcaagaacaacggcggggccgcagccatggagaagctcagctccatcaaatcccaaatgatttatgagatcattgataattctcaaggattttatgtatgcccagtggagcgccagaatagaagcaggatgaacatcccatttcgcattggcaacgccaaaggagacgaagctttggaaaagcggtttcttgacaaggcggtagaactcaacatgatctccttgaaggggcacaggtcagtgggaggcattcgtgcctctctgtataacgctgtcacaaccgaagacgttgagaagctggcggccttcatgaagaatttcttggagatgcatcagctgtga
Luc (firefly)	Atggccgatgctaagaacattaagaagggccctgctcccttctaccctctggaggatggcaccgctggcgagcagctgcacaaggccatgaagaggtatgccctggtgcctggcaccattgccttcaccgatgcccacattgaggtggacatcacctatgccgagtacttcgagatgtctgtgcgcctggccgaggccatgaagaggtacggcctgaacaccaaccaccgcatcgtggtgtgctctgagaactctctgcagttcttcatgccagtgctgggcgccctgttcatcggagtggccgtggcccctgctaacgacatttacaacgagcgcgagctgctgaacagcatgggcatttctcagcctaccgtggtgttcgtgtctaagaagggcctgcagaagatcctgaacgtgcagaagaagctgcctatcatccagaagatcatcatcatggactctaagaccgactaccagggcttccagagcatgtacacattcgtgacatctcatctgcctcctggcttcaacgagtacgacttcgtgccagagtctttcgacagggacaaaaccattgccctgatcatgaacagctctgggtctaccggcctgcctaagggcgtggccctgcctcatcgcaccgcctgtgtgcgcttctctcacgcccgcgaccctattttcggcaaccagatcatccccgacaccgctattctgagcgtggtgccattccaccacggcttcggcatgttcaccaccctgggctacctgatttgcggctttcgggtggtgctgatgtaccgcttcgaggaggagctgttcctgcgcagcctgcaagactacaaaattcagtctgccctgctggtgccaaccctgttcagcttcttcgctaagagcaccctgatcgacaagtacgacctgtctaacctgcacgagattgcctctggcggcgccccactgtctaaggaggtgggcgaagccgtggccaagcgctttcatctgccaggcatccgccagggctacggcctgaccgagacaaccagcgccattctgattaccccagagggcgacgacaagcctggcgccgtgggcaaggtggtgccattcttcgaggccaaggtggtggacctggacaccggcaagaccctgggagtgaaccagcgcggcgagctgtgtgtgcgcggccctatgattatgtccggctacgtgaataaccctgaggccacaaacgccctgatcgacaaggacggctggctgcactctggcgacattgcctactgggacgaggacgagcacttcttcatcgtggaccgcctgaagtctctgatcaagtacaagggctaccaggtggccccagccgagctggagtctatcctgctgcagcaccctaacattttcgacgccggagtggccggcctgcccgacgacgatgccggcgagctgcctgccgccgtcgtcgtgctggaacacggcaagaccatgaccgagaaggagatcgtggactatgtggccagccaggtgacaaccgccaagaagctgcgcggcggagtggtgttcgtggacgaggtgcccaagggcctgaccggcaagctggacgcccgcaagatccgcgagatcctgatcaaggctaagaaaggcggcaagatcgccgtgtaa
YAP1 (mice)	Atggagcccgcgcaacagccgccgccccagccggccccgcaaggccccgcgccgccgtccgtgtctccggccgggacccccgcggccccgcccgcacccccggccggccaccaggtcgtgcacgtccgcggggactcggagaccgacttggaggcgctcttcaatgccgtcatgaaccccaagacggccaacgtgcctcagaccgtgcccatgcggcttcgcaagctgcccgactccttcttcaagccgcctgagcccaagtcccactcgcgacaggccagtactgatgcaggtactgcgggagctctgactccacagcatgttcgagctcactcctctccagcctccctgcagctgggtgccgtttctcctgggacactcacagccagtggcgttgtctctggccctgccgctgcccctgcagctcagcatctccggcagtcctcctttgagatccctgatgatgtaccactgccagcaggctgggagatggccaagacatcttctggtcaaagatacttcttaaatcacaacgatcagacaacaacatggcaggacccccggaaggccatgctttcgcaactgaacgttcctgcgcctgccagcccagcggtgccccagacgctgatgaattctgcctcaggacctcttcctgatggatgggagcaagccatgactcaggatggagaagtttactacataaaccataagaacaagaccacatcctggctggacccaaggctggaccctcgttttgccatgaaccagaggatcactcagagtgctccagtgaagcagcccccacccttggctccccagagcccacagggaggcgtcctgggtggaggcagttccaaccagcagcagcaaatacagctgcagcagttacagatggagaaggagagactgcggttgaaacaacaggaattatttcggcaggcaatacggaatatcaatcccagcacagcaaatgctccaaaatgtcaggaattagctctgcgcagccagttgcctacactggagcaggatggagggactccgaatgcagtgtcttctcctgggatgtctcaggaattgagaacaatgacaaccaatagttccgatccctttcttaacagtggcacctatcactctcgagatgagagcacagacagcggcctcagcatgagcagctacagcatccctcggaccccagacgacttcctcaacagtgtggatgaaatggatacaggagacaccatcagccaaagcaccctgccgtcacagcagagccgcttccccgactacctggaagccctccctgggacaaatgtggaccttggcacactggaaggagatgcaatgaacatagaaggggaggagctgatgcccagtctgcaggaagcgctgagttccgaaatcttggacgtggagtctgtgttggctgccaccaagctagataaagaaagctttctcacgtggttatag

**Table 2:** ** Antibodies used in this study.** Here is the list of antibodies used in this study for immunostaining and western blot analysis.

Antigen	Dilution	Company	Catalog number
PSAT1	1:100	Invitrogen	PA5-22124
BrdU	1:200	Abcam	ab6326
α-Actinin	1:100	Abcam	ab9465
Ki67	1:100	Abcam	ab16667
pH3	1:100	Millipore	06-570
Troponin I	1:50	Santa Cruz Biotechnology	SC-15368
GFP	1:500	Abcam	ab13970
CD31	1:100	R&D Biosystems	AF3628
OHG	1:100	Abcam	ab62623
WGA	1:50	Life technology	W11261
β-catenin	1:200	Cell signaling	9582
DsRed	1:200	Living Colors	632496
Troponin I	1:200	Abcam	Ab47003
Troponin T	1:200	Invitrogen	MA5-12960
CD45	1:200	R&D Biosystems	AF114
Vimentin	1:100	Abcam	Ab24525
GAPDH	1:1000	Cell signaling	8884

**Table 3:** ** Primer sequences for qPCR in this study.** Here is the list of primers used in this study for qPCR analysis of different gene expression.

Gene	Forward	Reverse
PSAT1	caagcacctggaacctcaac	caccagcgaaaatcacacca
cMyc	aggcagctctggagtgagag	cctggctcgcagattgtaag
Cdc20	ttcgtgttcgagagcgatttg	accttggaactagatttgccag
Cdk1	tttcggccttgccagagcgtt	gtggagtagcgagccgagcc
Ccnd2	gtcacccctcacgacttcat	ttccagttgcaatcatcgac
Ccnb1	aaggtgcctgtgtgtgaacc	gtcagccccatcatctgcg
18s	agtccctgccctttgtacaca	cgatccgagggcctcacta
P27	aggagagccaggatgtcagc	cagagtttgcctgagacccaa
P21	gacaagaggcccagtacttc	gcttggagtgatagaaatctgtc
TGFβ1	cctgtccaaactaaggc	ggttttctcatagatggcg
Cola1	ctggcaagaagggagatga	caccatccaaaccactgaaa
Cola2	aggtcttcctggagctgatg	ccccacagggccttctttac
FN1	aggaagccgaggttttaactg	aggacgctcataagtgtcacc
Vegfa	aggctgctgtaacgatgaag	tctcctatgtgctggctttg
Vegfb	ggagaagagtggagcacagg	atggcagctctgggagataa
FGF2	aagcggctctactgcaagaacg	ccttgatagacacaactcctctc

**Table 4:** ** Primer sequences for ChIP-qPCR study.** Here is the list of ChiP-primers used in this study to analyze transactivation of PSAT1 by YAP1.

Gene	Forward	Reverse
Negative control	aggcacataggaggtaaaagttc	ggaggtcacaggaggacttc
Positive control (Cand1-126)	ttggttctgtcggatgtctg	ttggttctgtcggatgtctg
rPSAT1-558	catcctcccgagtgagtaatg	gggtgaagaaagggctaaag
rPSAT1-464	catttgttccagagccagtc	ttctttgggtgccttgtctc
rPSAT1-836	ccaggacagccactctcaag	gcacaagagccaagattcag

## Abbreviations

ModRNA: modified mRNA
MI: myocardial infarction
CVD: cardiovascular disease
HF: heart failure
CM: cardiomyocyte
PSAT1: phosphoserine aminotransferase 1
Echo: echocardiography
EF: ejection fraction
FS: fraction shortening
NRVM: neonatal rat cardiomyocyte
SSP: serine synthesis pathway
MADM: mosaic analysis with double markers
GSH: glutathione
WGA: wheat germ agglutinin
LVIDd: left ventricular internal diameter
LVIDs: left ventricular internal diameter end-systole
LVPWd: end-diastolic left ventricular posterior wall thickness
LVPWs: end-systolic left ventricular posterior wall thickness
3PHP: 3-phosphohydroxy-pyruvate
3PS: phosphoserine
ROS: reactive oxygen species
PHGDH: phosphoglycerate dehydrogenase
PSPH: phosphoserine phosphatase
