# Supplementary material for: Phosphoserine aminotransferase 1 promotes serine synthesis pathway and cardiac repair after myocardial infarction
Source: Theranostics. 2025 Jun 18;15(15):7219–41. doi: 10.7150/thno.112077 (PMC12315692; doi:10.7150/thno.112077)
Supplement: Supplementary file 1 — Supplementary figures. [file thnov15p7219s1.pdf]

Supplemental Information:

Supplementary Figures and Figure Legends

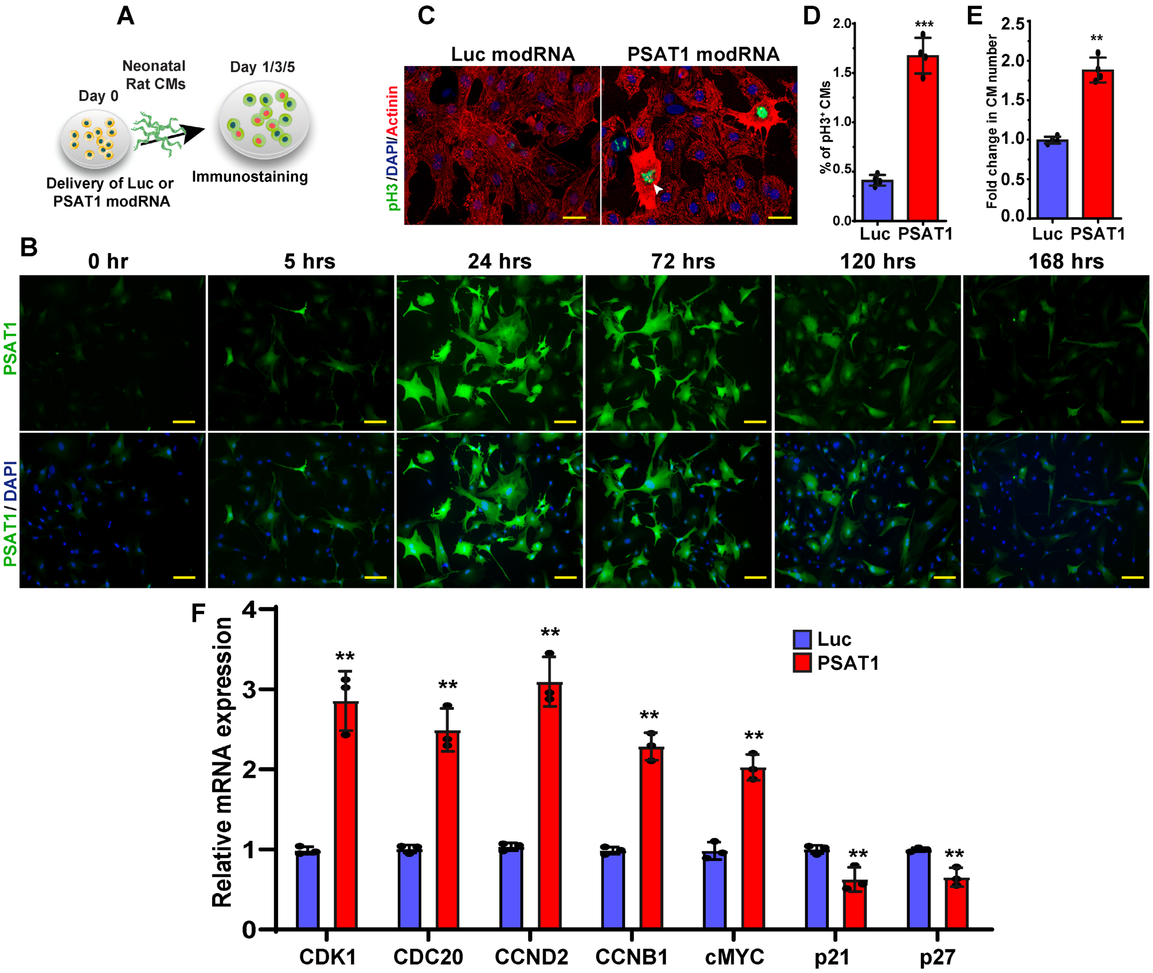

Supplementary Figure S1. PSAT1 modRNA induces cardiomyocyte cell cycle *in vitro*. A. Experimental scheme for PSAT1 modRNA expression in NRVM and its effect on CM cell cycle *in vitro*. **B.** Representative images of PSAT1 expression at different time points post-transfection of PSAT1 modRNA, Green ($PSAT1^{+}$), and DAPI-nucleus marker, showing transient nature of modRNA expression platform (n=4 (4 different cell isolations)). **C.** Representative images of PSAT1-induced CM mitosis analyzed by mitosis marker (pH3) expression (green), CM-specific marker α-sarcomeric $actinin^{+}$ (Red), and DAPI (blue). **D.** Quantification of $PH3^{+}$ CMs in D (n = 4 (4 different cell isolations)). **E.** Quantification of PSAT1 induced CM number analysis 5 days post-transfection of Luc or PSAT1 modRNA expression, showing PSAT1 induce CM mitosis in NRVMs (n = 4 (4 different cell isolations)). **F.** The m-RNA expression of cell-cycle-promoting genes or cell-cycle inhibitors after PSAT1 or Luc modRNA expression in NRVMs (n = 3 (3 different cell isolations)). Unpaired two-tailed t-test for D-E, F ***, P < 0.001, **, P < 0.01. Scale bar = 50 μm (B), 20 μm (D).

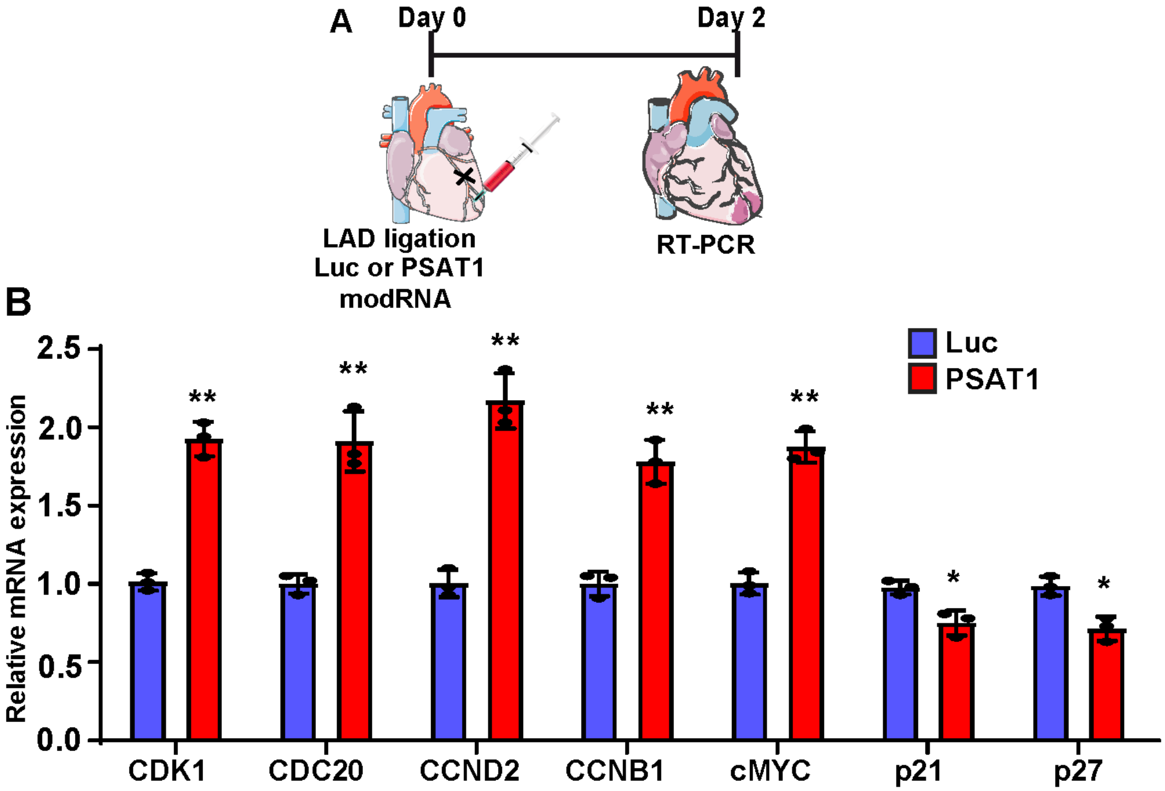

Supplementary Figure S2. PSAT1 modRNA regulates cardiomyocyte cell cycle genes *in vivo*. A. Experimental plan for the effect of PSAT1 or Luc modRNA delivery on cell cycle marker expression 2 days post-MI. **B.** mRNA expression of cell-cycle-promoting genes or cell-cycle inhibitors after PSAT1 or Luc modRNA delivery post-MI (n = 3). Unpaired two-tailed t-test for B **, P < 0.01, *, P < 0.1.

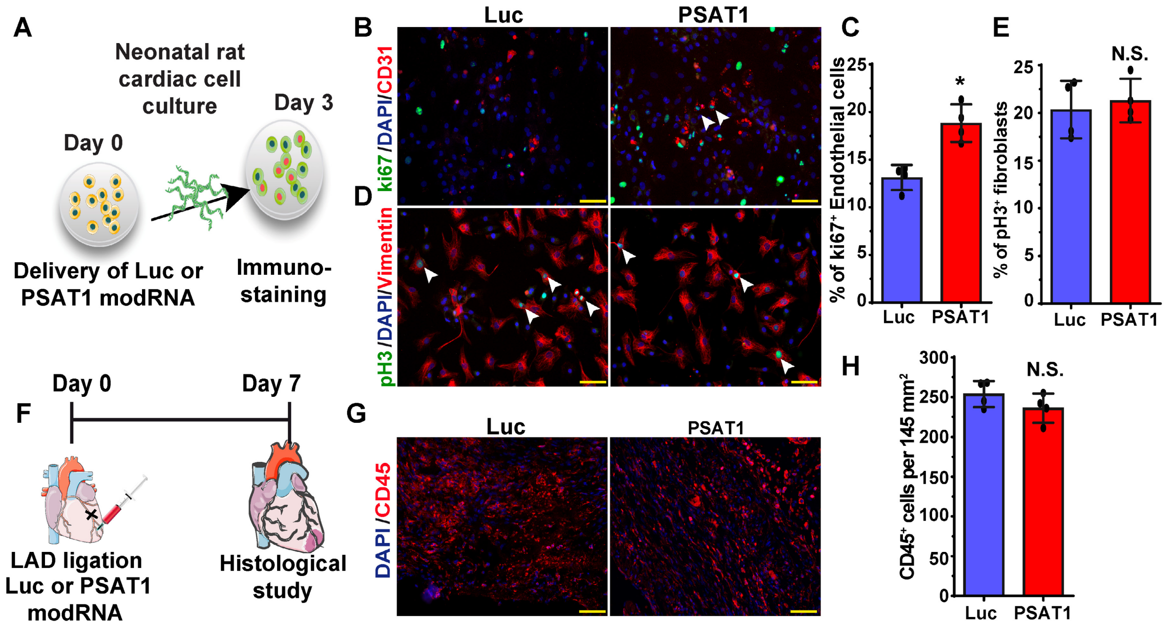

Supplementary Figure S3. The role of PSAT1 modRNA on endothelial cell, fibroblast and immune cell proliferation. A. Experimental scheme for PSAT1 modRNA expression in neonatal rat cardiac cell culture (mixed cell culture) and its effect on endothelial and fibroblast proliferation *in vitro*. **B.** Representative images of PSAT1-induced endothelial cell proliferation analyzed by cell cycle marker (ki67) expression (green), endothelial cell-specific marker $CD31^+$ (Red), and DAPI (blue). **c.** Quantification of $ki67^+$ endothelial cells in B (n = 4 (4 different cell isolations)). **D.** Representative images of PSAT1-induced fibroblast proliferation analyzed by cell cycle marker (pH3) expression (green), fibroblast marker $Vimentin^+$ (Red), and DAPI (blue). **E.** Quantification of $pH3^+$ fibroblasts in D (n = 4 (4 different cell isolations)). **F.** Experimental plan for the effect of PSAT1 or Luc modRNA delivery on $CD45^+$ (immune cells) 7 days post-MI. **G.** Representative images of Luc or PSAT1 modRNA-effect on immune cell numbers analyzed by immune cell marker CD45 (Red), and DAPI (blue). **H.** Quantification of immune cell numbers in G (n = 5). Unpaired two-tailed t-test for C, E, H. ***, $P < 0.001$, **, $P < 0.01$. Scale bar = 50 µm (B, D and G).

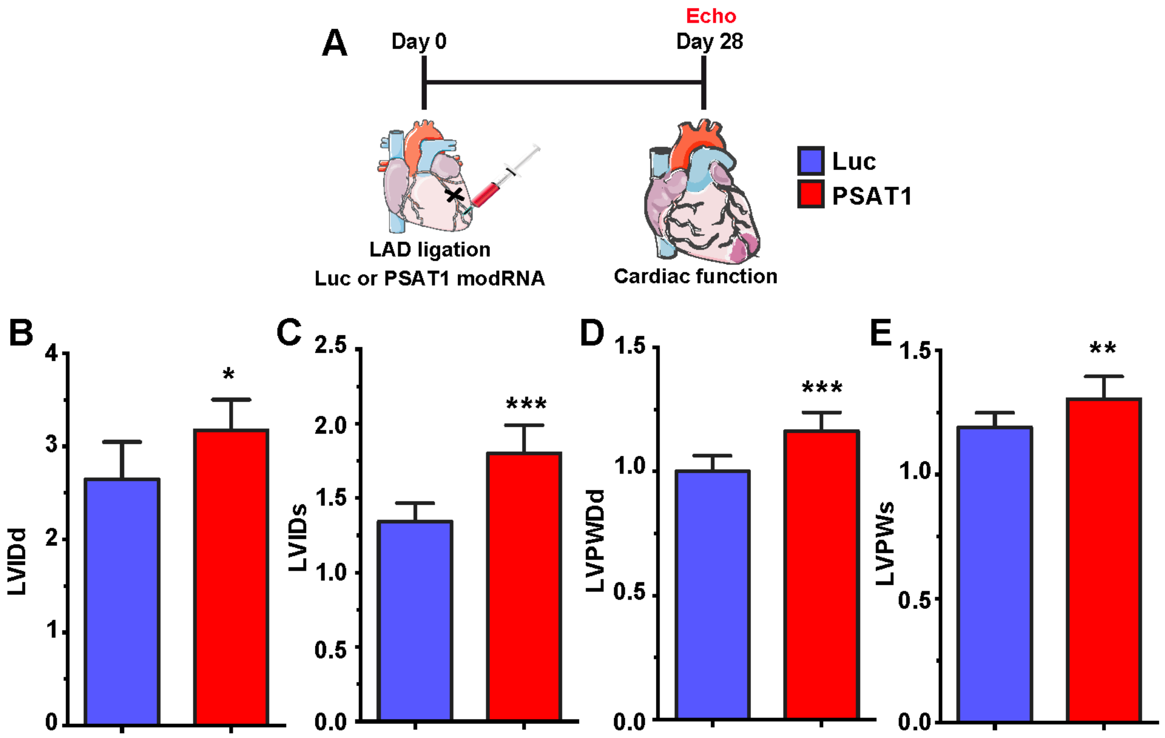

Supplementary Figure S4. Myocardial delivery of PSAT1 modRNA improves cardiac function post-MI. A. Experimental timeline used for evaluating the effect of PSAT1 or Luc modRNA delivery on cardiac function and outcome in a mouse MI model. **B-C.** Echocardiography was used to evaluate left ventricular internal diameter end-diastole (28 days post-MI) (LVIDd) or end-systole (LVIDs) 28 days post-Luc or PSAT1 modRNA delivery to the heart post-MI (Luc modRNA, n = 8; PSAT1 modRNA, n = 9). **D-E**. End-diastolic left ventricular posterior wall thickness (LVPWd) or end-systolic left ventricular posterior wall thickness (LVPWs) in the PSAT1, or Luc modRNA injected mice (28 days post-MI) (Luc modRNA, n = 8; PSAT1 modRNA, n = 9). Unpaired two-tailed t-test for B-E. ***, $P < 0.001$, **, $P < 0.01$, *, $P < 0.1$, N.S, Not Significant.

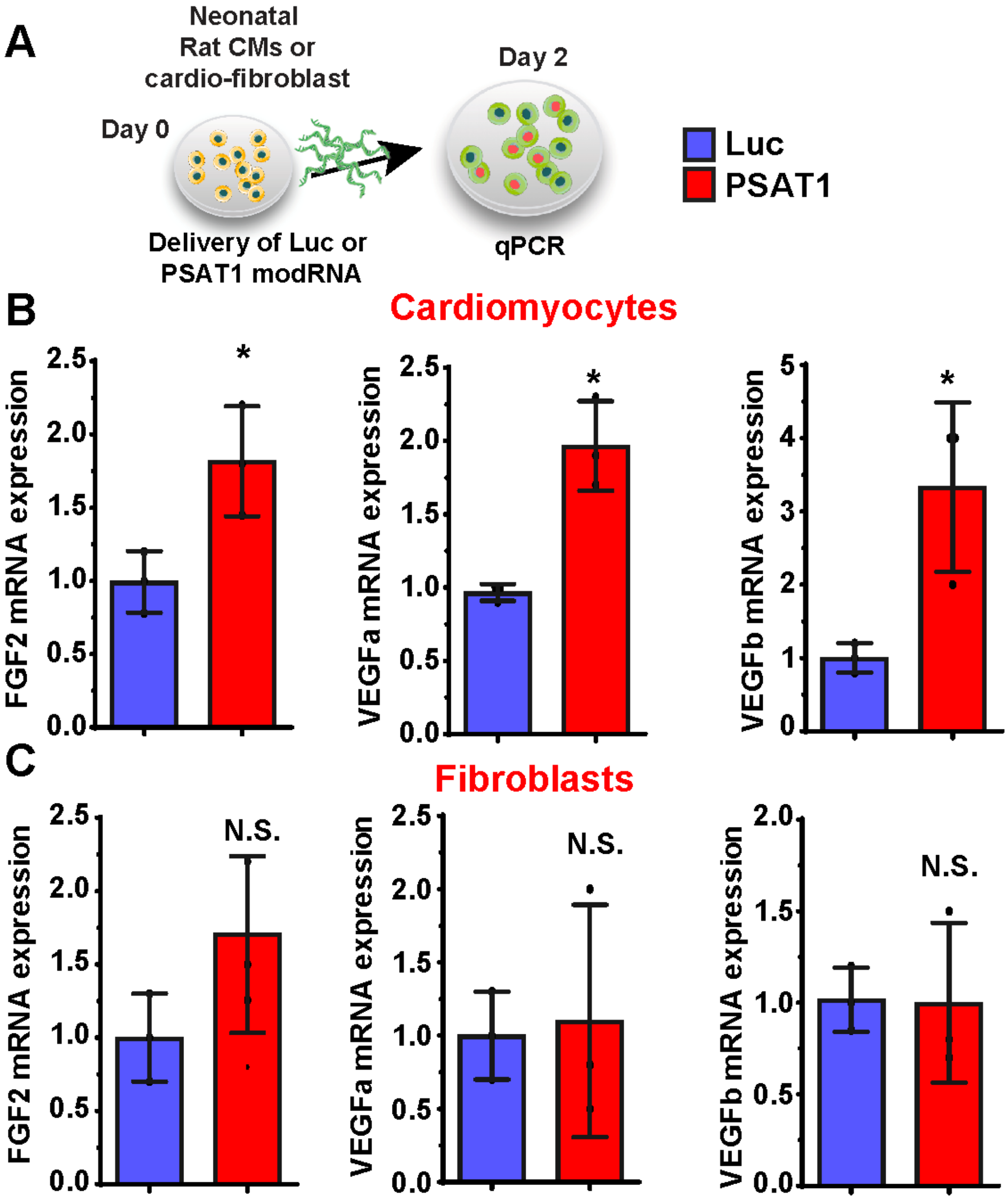

Supplementary Figure S5. PSAT1 modRNA regulates the expression of angiogenesis markers in neonatal rat (NRVMs) and fibroblasts. A. Experimental scheme for PSAT1 modRNA expression in NRVM or fibroblasts and its effect on expression of FGF2, VEGFa and VEGFb *in vitro* in CMs and fibroblast. **B.** Quantitative analysis of FGF2, VEGFa and VEGFb mRNA expression in CMs in (B) or in fibroblast in (C) after PSAT1 or Luc modRNA transfection (n = 3 (3 different cell isolations)). Unpaired two-tailed t-test for B and C. *, $P < 0.1$, N.S, Not Significant.

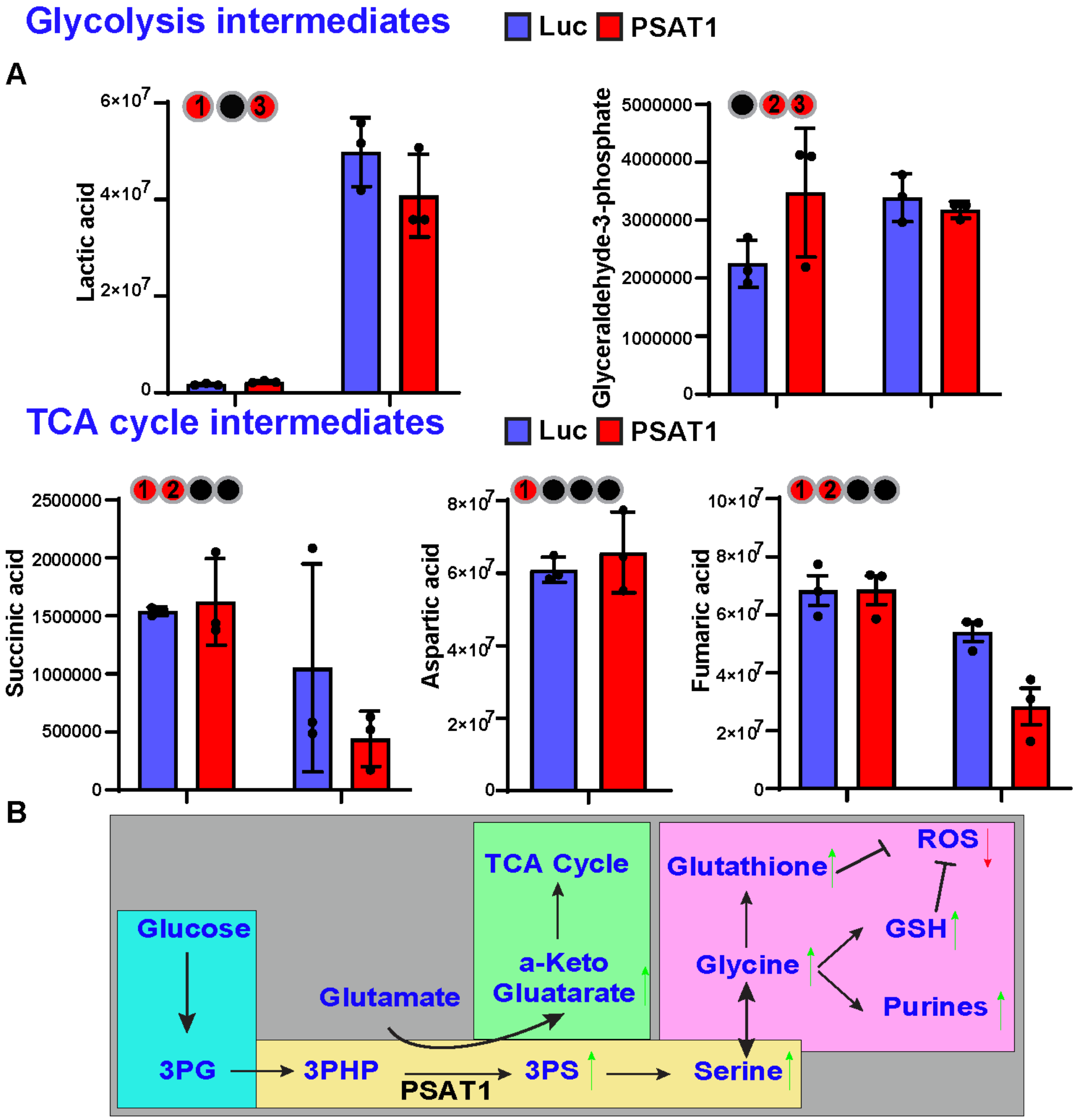

Supplementary Figure S6. PSAT1 modRNA delivery into CMs redirects glucose carbon flow into serine and nucleotide synthetic pathways *in vitro*. A. We analyzed absolute intracellular labeled metabolites with [U-^{13}C] glucose flux using mass spectrometry in P2-P3 NRVMs transfected with PSAT1 or Luc modRNA. The levels of [U-^{13}C]-labeled glycolysis metabolites were evaluated 10 min after [U-^{13}C] glucose addition, TCA metabolites were evaluated 6 h. after [U-^{13}C] glucose addition and serine and nucleotides synthesis pathway metabolites were evaluated 24 h after [U-^{13}C] glucose addition (n = 3). In all panels, the X-axis represents the carbon number in the given structure of a specific molecule and the Y-axis represents absolute intensity. **B.** Proposed PSAT1 induced metabolic pathway in neonatal CMs. Unpaired two-tailed t-test. ****, $P < 0.0001$, **, $P < 0.01$, *, $P < 0.1$, N.S, Not Significant.

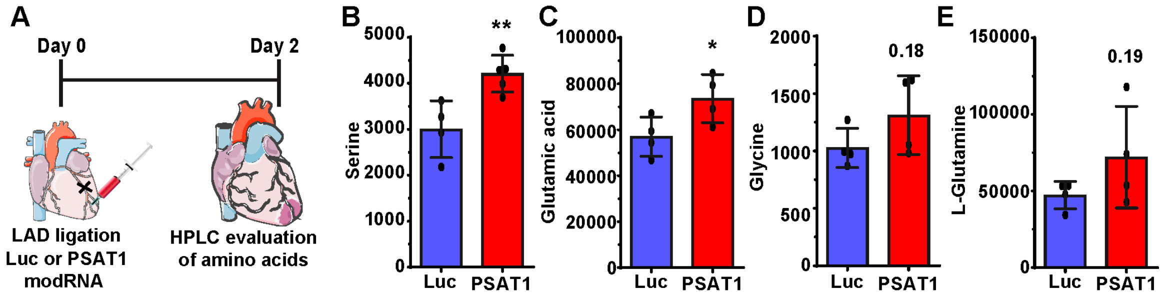

Supplementary Figure S7. PSAT1 modRNA induces serine levels in mice heart post-MI. A. Experimental timeline used for evaluating the effect of PSAT1 or Luc modRNA delivery on SSP related metabolite levels post-MI and modRNA delivery. **B-E**. Serine (B), glutamic acid (C), glycine (D) and L-glutamine (E) levels of mice heart samples were analyzed using HPLC (n = 4) 2 days post-MI and modRNA delivery. The data there is induction in SSP pathway post-MI and PSAT1 modRNA delivery. Unpaired two-tailed t-test. ***, P < 0.001, **, P < 0.01.

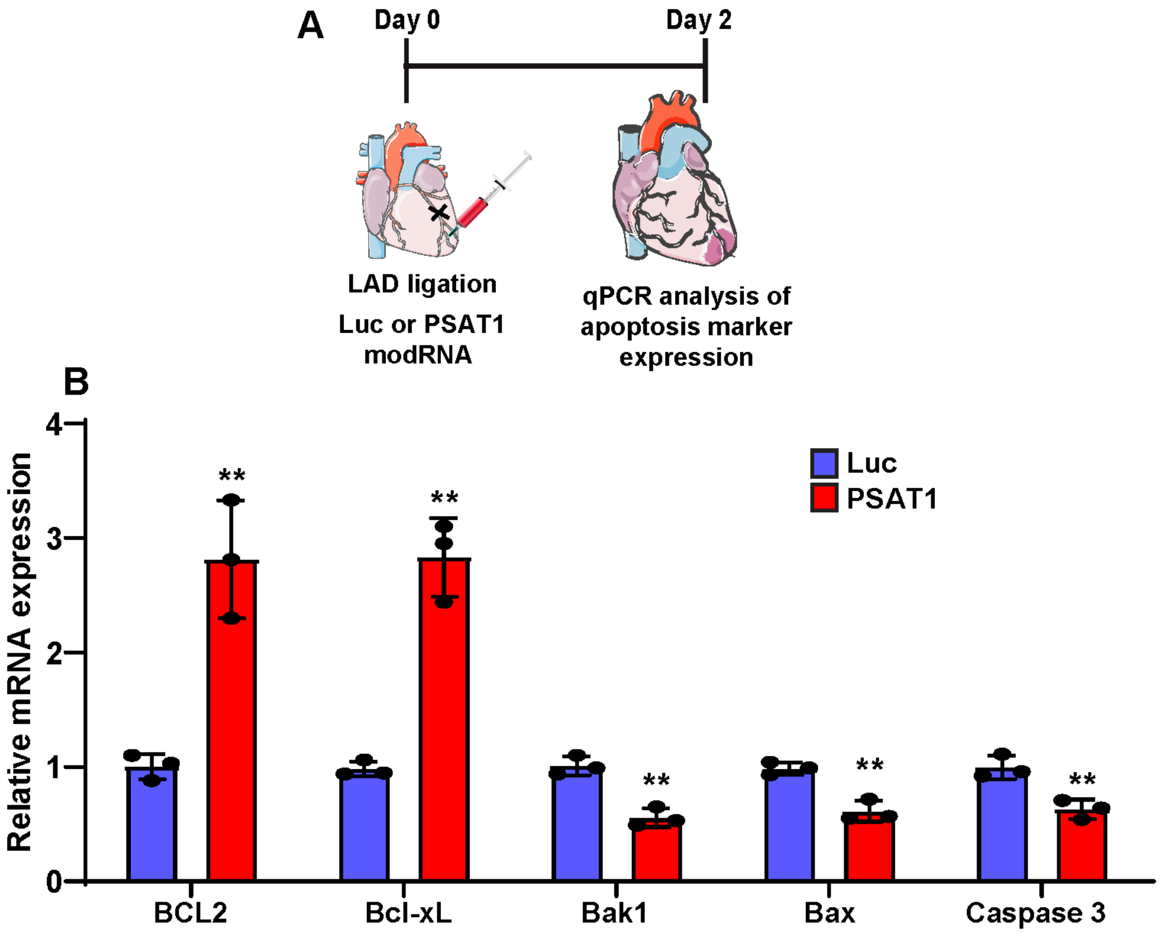

Supplementary Figure S8. PSAT1 modRNA regulates apoptosis marker gene expression *in vivo*. A. Experimental plan for the effect of PSAT1 or Luc modRNA delivery on apoptosis marker expression 2 days post-MI. **B.** Apoptosis marker gene (BCL2, Bcl-xL, Bak1, Bax and Caspase 3) expression after PSAT1 or Luc modRNA delivery post-MI (n = 3). Unpaired two-tailed t-test. **, $P < 0.01$.

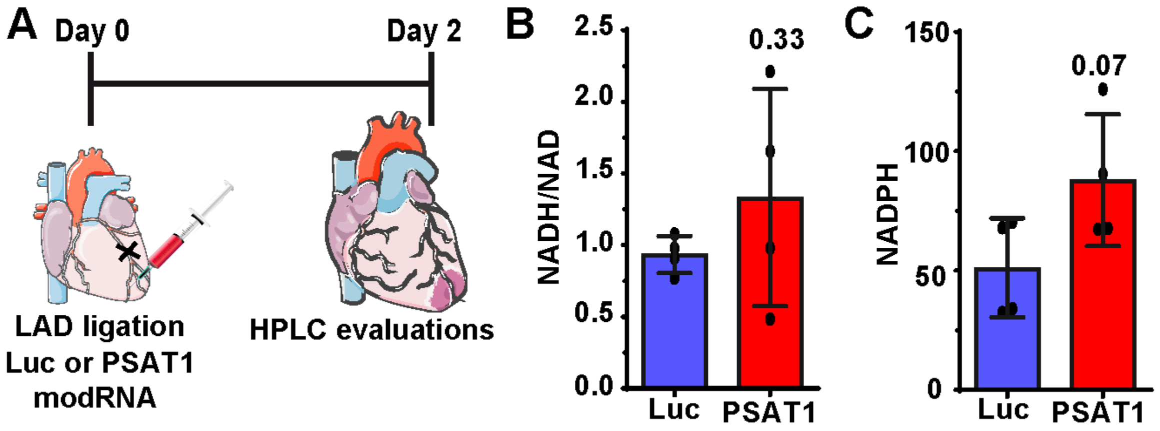

Supplementary Figure S9. PSAT1 modRNA inhibits oxidative stress post-MI. **A.** Experimental timeline used for evaluating the effect of PSAT1 or Luc modRNA delivery on the levels of reducing agent NADH, NAD and NADPH. **B-C**. The ratio of NADH/NAD (B) and NADPH levels (C) post-MI samples were analyzed using HPLC (n = 4). Unpaired two-tailed t-test for B-C.

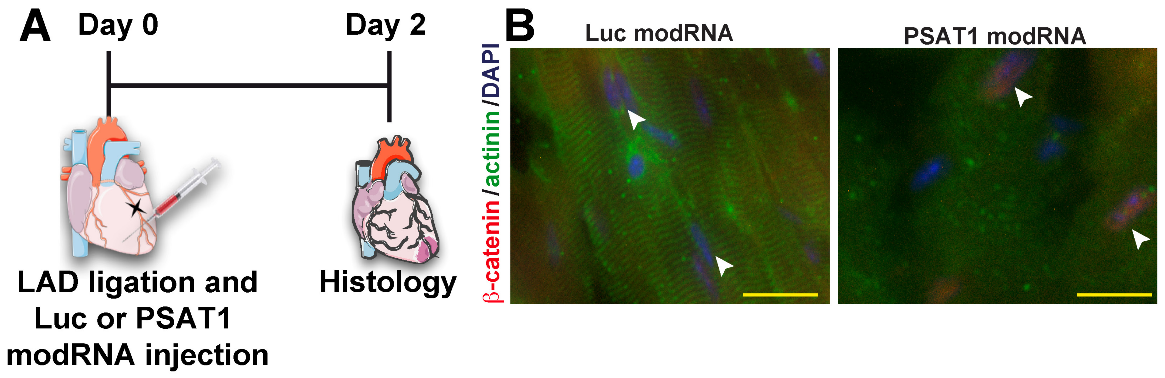

Supplementary Figure S10. PSAT1 modRNA induces translocation of β-catenin to nucleus post-MI. A. Experimental scheme for modRNA expression (Luc Vs PSAT1) in mouse heart post-MI and β-catenin immunostaining. **B.** Representative images of β-catenin expression 2 days post-MI and transfection of Luc or PSAT1 modRNA, Red (β-catenin$^+$), Green–CM-specific marker (α-sarcomeric actinin$^+$), and DAPI-nucleus marker (n = 4). The nuclear transfer of β-catenin suggests activation and stabilization of β-catenin in PSAT1 modRNA injected mice heart post-MI. Scale bar = 50 μm (B).
